# In silico analysis of pH stabilising mutations of hemagglutinin of influenza A virus H5N1 clade 2.3.4.4b

**DOI:** 10.1038/s44298-026-00236-y

**Published:** 2026-09-25

**Authors:** Daniel Christian Lauster, Christian Sieben, Matthias Ballauff, Andreas Herrmann

**Affiliations:** 1 Freie Universität Berlin, Institut für Pharmazie, Biopharmazeutika, Berlin, Germany; 2 Nanoinfektionsbiologie (NIBI), HZI - Helmholtz-Zentrum für Infektionsforschung GmbH, Braunschweig, Germany; 3 Freie Universität Berlin, Institut für Chemie und Biochemie, SupraFAB, Berlin, Germany

**Keywords:** Biochemistry, Computational biology and bioinformatics, Microbiology

## Abstract

Highly pathogenic avian influenza A(H5N1) viruses are expanding their host range among mammals, raising concerns about their pandemic potential. Building on recently published deep mutational scanning data^1^, we show that hemagglutinin retains structural plasticity to increase acid stability through independent mechanisms, including modulation of electrostatic interactions, hydrogen-bonding networks and hydrophobic packing that may facilitate human adaptation. These findings illustrate how structural analyses can strengthen genomic surveillance for pandemic risk assessment.

## Introduction

The ongoing global circulation of the avian influenza A (H5N1) virus, particularly the clade 2.3.4.4b strain, has raised serious concerns about its potential to cause a future pandemic. While the mortality rate among humans has been lower than in previous H5N1 outbreaks^2^, the virus’s unprecedented spread across wild and domestic birds, its ability to infect multiple mammalian species and the evidence of mammal-to-mammal transmission underscore its zoonotic potential. Its ability to replicate efficiently in mammalian hosts such as dairy cattle, producing high viral loads in milk, indicates ongoing adaptation that could facilitate human infection. Therefore, understanding the biological barriers that still limit efficient human transmission is essential for pandemic preparedness.

**Fig. 1:**
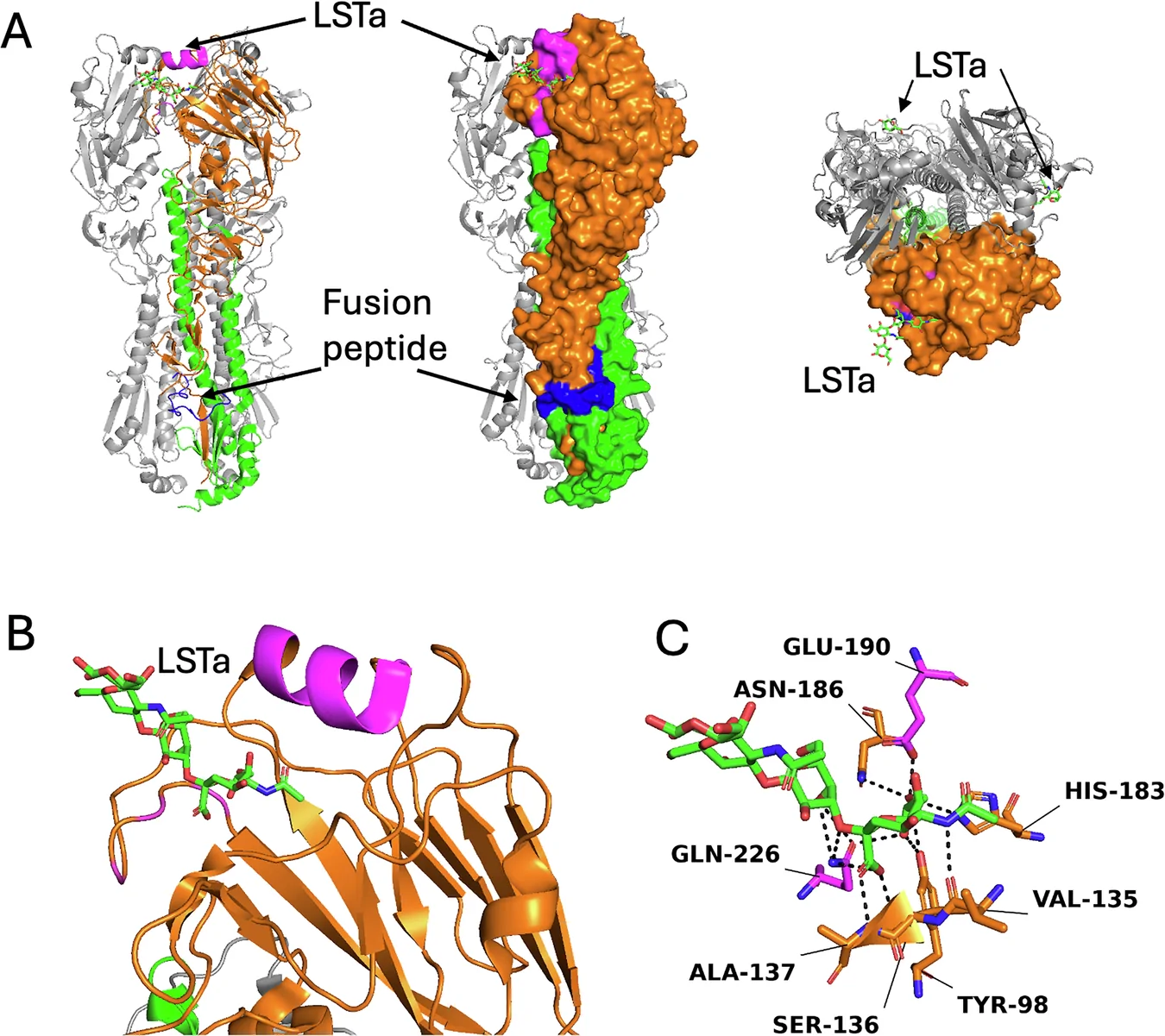
The trimeric structure of the hemagglutinin ectodomain (ECD) of H5 A/Texas/37/2024 (pdb 9DIP41) with the avian receptor analogue Sialyllacto-N-tetraose a (LSTa; α(2,3)-Sia link). **A** Left: The HA1 subunit (orange) and the HA2 subunit (green) are shown for one monomer. The helix of the binding pocket is shown in magenta, the fusion peptide in blue. The other two monomers are in grey. Middle: Surface of the HA1 subunit. Right: Top view. **B** LSTa and its HA binding pocket are enlarged. **C** Residues of HA1 with polar interaction with sialic acid (dashed lines) are shown (see text). The interactions are in the range of 2.5–3.4 Å.

Human infection by avian influenza viruses is usually ineffective due to several host barriers, such as suboptimal receptor recognition and incompatibility with the host cell’s machinery. The major homotrimeric organized spike glycoprotein hemagglutinin (HA) is a key determinant of host specificity being responsible for receptor binding and membrane fusion^3^. Mutations that modify its receptor affinity or structural stability can profoundly influence host adaptation and transmissibility. Each HA monomer (consisting of around 500 amino acids) is cleaved into HA1 and HA2 subunits (Fig. 1). In highly pathogenic avian influenza viruses (HPAIVs) such as H5N1, the polybasic cleavage site enables activation by furin throughout the body, resulting in systemic infectivity in birds^4,5^.The globular head of HA1 binds to host receptors, while HA2 forms a stem in the trimer that anchors the HA trimer to the viral envelope. The HA2 stem region is a central determinant of the prefusion metastable conformation and is highly conserved among IAV subtypes. Its N-terminus forms the 20 amino acid long hydrophobic fusion peptide^6–8^. Upon endocytic uptake of the virus, the acidification of the endosomal lumen triggers an irreversible conformational change in the HA, exposing the fusion peptide. This triggers fusion between the virus and the endosome, thereby releasing the segmented viral genome.

The prefusion, neutral pH form of the HA trimer is metastable - stable enough to persist during transmission, yet ready to refold in response to endosomal acidification. If the HA trimer is too unstable, it may lose its fusogenic potential prematurely; conversely, if it is too stable, fusion may fail. Therefore, optimal acid stability and pH-dependent activation are crucial for viral infectivity. In wild aquatic birds - the natural reservoir of avian IAV - acid stability is essential for persistence in water^9,10^. Transition to poultry is accompanied by the acquisition of the polybasic cleavage site and reduced acid stability^11,12^. Adaptation to mammals typically requires further stabilisation and a lower fusion-triggering pH (from ~5.7–6.0 in avian viruses to ~5.0–5.5 in human strains)^13–17^, enhancing survival during airborne transmission and passage through the mildly acidic human respiratory tract. Conversely, premature triggering of the conformational change in transmission droplets or early endosomes can inactivate the virus infectivity^17–21^.

The emergence of H5N1 clade 2.3.4.4b exemplifies this adaptive trajectory. It likely arose around 2020 through reassortment between a highly pathogenic avian H5N8 strain and a low-pathogenic avian virus in Central Asia or Europe^22–24^, and has since spread globally. It has caused high mortality in both domestic and wild birds, and expanded its host range to include mammals^25^. In 2024, sustained mammal-to-mammal transmission occurred among US dairy cattle, with replication in mammary glands and high viral titres in milk^26^. Genomic analyses identified variants such as genotype B3.13, which carries PB2 polymerase mutations associated with mammalian adaptation^27–31^, heightening concerns about the potential for zoonotic transmission. Nevertheless, the HA of clade 2.3.4.4b still primarily binds avian-type α2,3-linked sialic acid receptors and shows only weak affinity for human-type α2,6-linked receptors^32–36^. Experimental studies indicate that few HA mutations can shift receptor specificity and enhance acid stability, enabling airborne transmission in mammals such as ferrets^18,31,37–40^. Substitutions including Gln226Leu, Asn224Lys, and Gly228Ser in the receptor-binding pocket increase α2,6-linked sialic acid affinity - a prerequisite for human adaptation^34,35,41–45^, yet remain rare in circulating 2.3.4.4b strains, suggesting efficient human adaptation requires multiple coordinated changes^35,36,46^. Moreover, stabilisation of the HA ectodomain at low pH appears essential for airborne transmissibility under the acidic conditions of respiratory droplets^17,18,37,47^. HA stability and function are governed by electrostatic interactions that stabilise the prefusion HA structure at neutral pH and regulate acidification-induced conformational changes^12,48–51^. In cleaved HA, protonation of surface-exposed ionisable residues lacking charged partners can destabilise the protein and shift the pH threshold for fusion triggering, thereby affecting membrane fusion efficiency and viral infectivity^52–54^ In summary, the efficient human-to-human transmission of H5N1 clade 2.3.4.4b will likely require concurrent adaptation via mutations in three key viral properties: polymerase activity, receptor-binding specificity, and HA stability.

This study examines the acid stability and structural adaptation of the hemagglutinin ectodomain (ECD) of H5N1 clade 2.3.4.4b, focusing on mutations that may enhance stability at low pH and thereby promote adaptation to human hosts. Building on the comprehensive analysis of ECD stability in circulating H5N1 viruses by Dadonaite et al. (2024)^1^, we explore the molecular determinants of enhanced acid stability and their potential contribution to mammalian adaptation.

Evidence from previous studies indicates that increased acid stability of the HA ectodomain facilitates adaptation to humans. We therefore examine how stabilizing mutations reshape the molecular architecture of the ECD, with particular emphasis on hydrogen-bonding networks, salt-bridge formation, and electrostatic surface potential. By integrating insights from previous mutational studies with computational structural analyses, we provide a mechanistic framework for understanding how enhanced acid stability may influence the evolutionary trajectory and zoonotic potential of H5N1 clade 2.3.4.4b.

## Methods

Dadonaite et al. (2024)^1^ used a lentivirus-based deep mutational scanning platform (Dadonaite et al., 2023)^55^ to analyse pseudoviruses encoding different HA mutants from A/American Wigeon/South Carolina/2021 (H5N1 clade 2.3.4.4b). They investigated the influence of these mutants on receptor specificity, HA stability and antibody escape. The cell entry of the pseudoviruses was measured using 293 T cells, which are derived from human embryonic kidney (HEK) 293 cells^1^. HA stability was assessed by measuring the retention of infectivity in 293 T cells following the incubation of the pseudoviruses in increasingly acidic conditions. Pre-incubating the viruses at an acidic pH as low as 5.3 leads to an irreversible conformational change in the HA, resulting in its inactivation. The pH at which this change occurs depends on the stability of the HA.

This study analyses the molecular consequences of the mutations identified by Dadonaite et al. (2024)^1^ that lead to an increase in the stability of the ECD of the HA of H5N1. A 3D structure of the ECD relevant for H5N1 of clade 2.3.4.4b serves as the basis. Although no 3D structure of the HA is available for the strain used by Dadonaite et al. (2024)^1^, a structure is available for H5 A/Texas/37/2024 (pdb 9DIP^41^), which serves as a reference strain for clade 2.3.4.4b^46^. This strain has caused numerous outbreaks of HPAI H5N1 in dairy cattle, affecting almost 300 herds in 14 US states^41^. According to the FluSurver (https://flusurver.bii.a-star.edu.sg; GISAID database) it shares 99.4% sequence identity with A/American Wigeon/South Carolina/2021, analysed by Dadonaite et al. (2024)^1^(see Scheme S1). The sequence differences were limited to three sites: Leu122Gln, Thr200Ile, and Asp240Asn. Figure 1 shows the 3D structure of the HA (pdb 9DIP^41^) with a natural sialo-pentasaccharide from human milk LSTa (α(2,3)-Sia link), which is an avian-type receptor analogue^56^.

The 3D crystal structure of HA (pdb 9DIP)^41^ was analysed using PyMOL (version 3.0.2; licence #51548) to predict the impact of mutations on polar interactions and surface potential. Polar interactions, including hydrogen bonds and salt bridges, were characterised and visualised. The influence of mutations on the local surface potential was calculated using the Adaptive Poisson-Boltzmann Solver (APBS) including PDB2PQR software (using AMBER Force Field) via the APBS server or PyMOL plugin^57,58^. The surface potential was calculated at 0.15 M NaCl (ionic radius: Na^+^ −1.08 Å; Cl^−^ −1.81 Å) for pH values of 5.3 and 7.4 in order to characterise the influence of mutations on the potential in the acidic and neutral range. In order to capture the full range of the potential in an accurate and differentiable manner, a false-code scaling of −5 kT/e (intense red) to +5 kT/e (intense blue) was selected. All lenghts of bonds shown in the figures are given in Å.

## Results

**Fig. 2:**
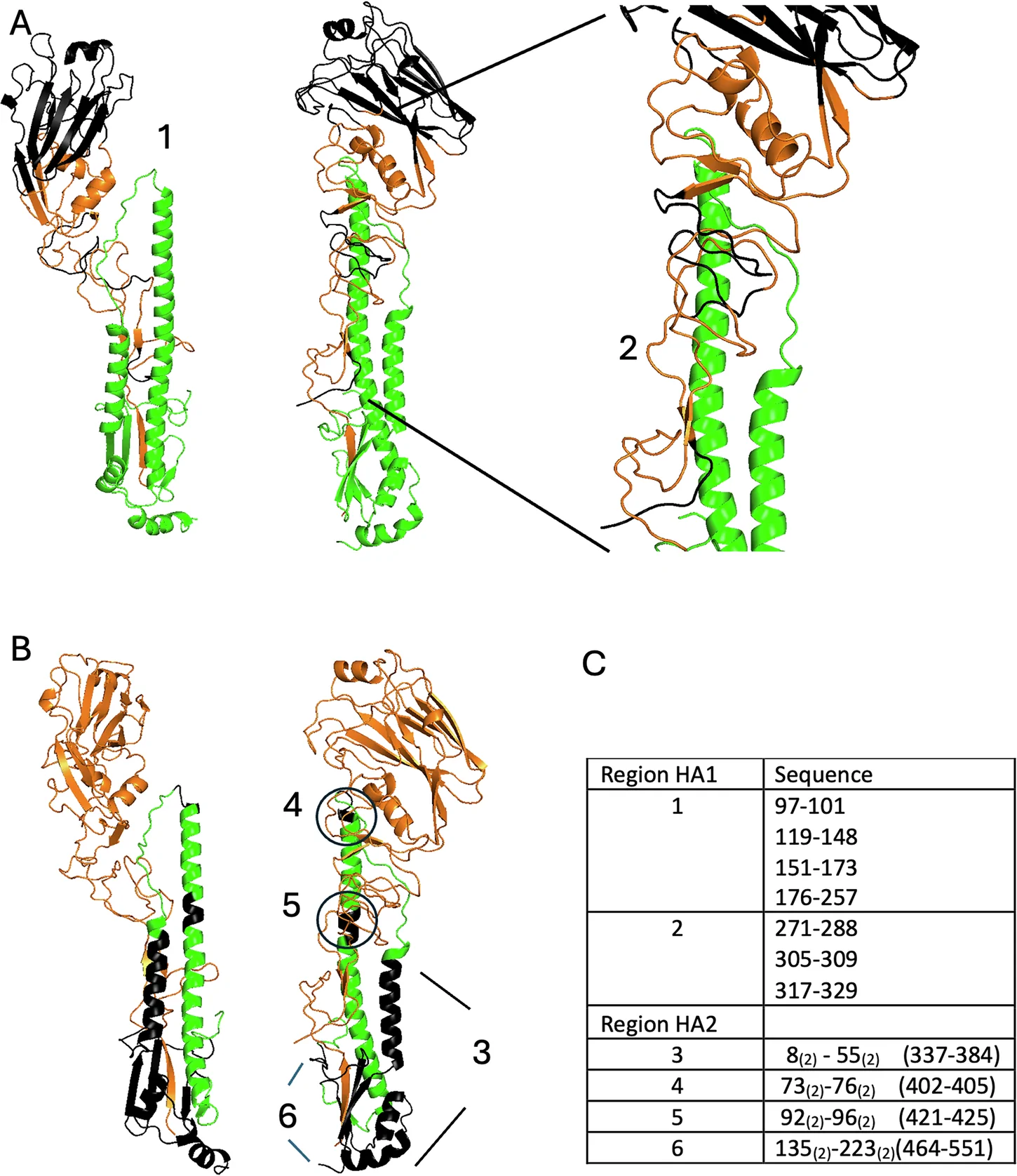
Sequence regions of the ECD in which mutations were consistently detrimental to stability. **A**, **B** HA Monomer of H5 A/Texas/37/2024 (pdb 9DIP^41^) with regions in which mutations were consistently detrimental to stability (shown in black) ((**A**) HA1, (**B**) HA2). **C** Sequence numbering of regions shown in black in (**A**, **B**).

Dadonaite et al. (2024)^1^ reported that their analysis revealed several stabilising mutations that were already known from previous studies to increase ECD stability: Tyr17His, Ala19Thr, Glu31Lys, His110Tyr, and Thr318Ile^9,13,18,59,60^ (see also our analysis below). Residues with a subscript index (2) are located in the HA2 subunit. HA1 residues have no index. H3 numbering is used throughout). Beyond these mutations, Dadonaite et al. highlighted additional mutations with a stabilising influence (see Fig. 4D in Dadonaite et al. (2024)^1^). Below, we address why these, and other mutations selected from the study by Dadonaite et al.’s (2024)^1^ could lead to HA stabilisation. It should be noted that there are larger sequence regions of the ECD in which mutations generally lead to HA trimer destabilisation (Fig. 2).

A significant portion of the HA1 head domain and extensive regions of the HA2 stem are characterised by these sequence regions in which mutations were consistently detrimental to stability. The stem region (HA2) is highly conserved because it is responsible for the metastable trimeric organisation of HA and the low pH-dependent conformational change that releases the fusion peptide. Although this study focuses on stabilising mutations, residues were analysed in Supplementary Information whose mutation has a detrimental effect on the stability of the ECD. (Figs. S1 to S9, Table S1).

**Fig. 3:**
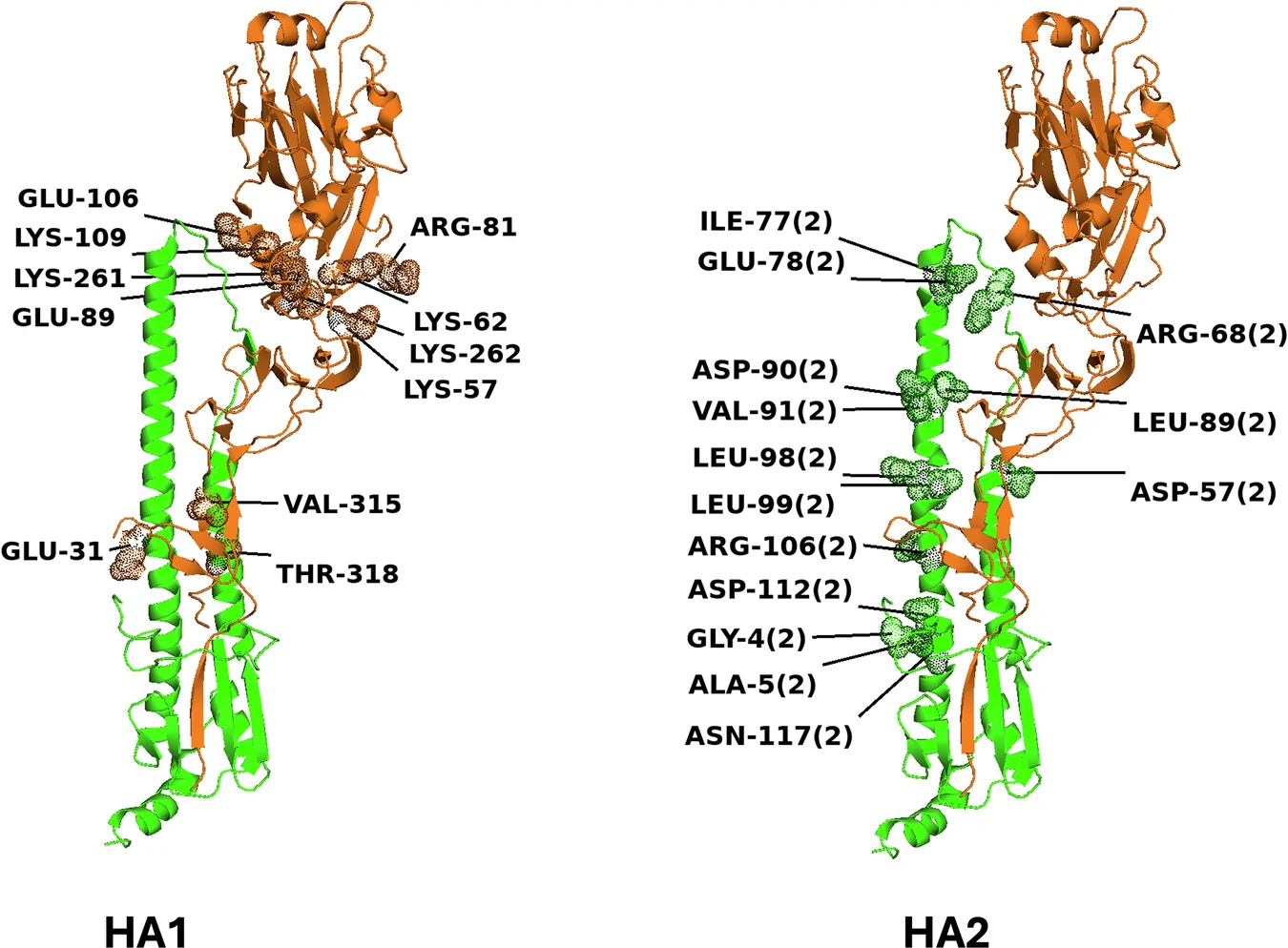
Stabilising mutations in the ECD of HA. Overview of the distribution of amino acid residues in HA1 and HA2 for which Dadonaite et al. (2024) identified mutations that stabilise the ECD.

Table 1 provides an overview of stabilising mutations, their influencing site, and if applicable, their influence on the surface potential of the ECD. The distribution of those mutations in the HA1 and HA2 subunits is shown in Fig. 3.

**Table 1:** Overview of stabilising mutations in the ectodomain and their location of influence, i.e., whether within the subunit, between the two subunits of a monomer and/or between monomers, as well as a possible influence on the surface potential of the trimer (indicated by √). For positions for which several stabilising amino-acid substitutions have been reported, X denotes different substitutions at the indicated residue

Sub-unit	Mutation	Influence of Mutation			
		Within subunit	Intramonomer HA1-HA2 interface	Intermonomer interface	Surface potential ectodomain
HA1	Glu31Lys	√			√
	Lys57Glu	√			√
	Lys62Glu	√			√
	Arg81Gly	√			√
	Arg90Glu	√			√
	Lys109Ala	√			√
	Lys109Gln	√			√
	Lys226Ile	√			√
	Lys269Glu	√			√
	Tyr17His		√		
	Glu106Asp		√		
	Val315X	√	√		
	Thr318Ile	√	√		
	His18Gln		√		
	His110X	√	√		√
	His184Arg	√		HA1-HA1	
HA2	Asp57_(2)_Tyr	√			√
	Lys58_(2)_Val			HA2-HA2	√
	Arg68_(2)_Ser		√		√
	Ile77_(2)_Leu			HA2-HA2	
	Glu78_(2)_Phe	√			
	Asp90_(2__)_X			HA2-HA2 HA2-HA1	
	Val91_(2)_Met			HA2-HA2	
	Asp95_(2)_X			HA2-HA2	
	Leu98_(2)_Met	√		HA2-HA2	
	L99_(2)_Met			HA2-HA2	
	Arg106_(2)_Glu			HA2-HA2	
	Asn117_(2)_X	√		HA1-HA2	√

### Stabilising mutations in the HA1 subunit

Residues within the HA1 subunit where mutations can stabilise the ectodomain are located essentially in the lower part of the head domain, above the stalk region, and in a small region of the ECD stalk.

#### Glu31Lys

**Fig. 4:**
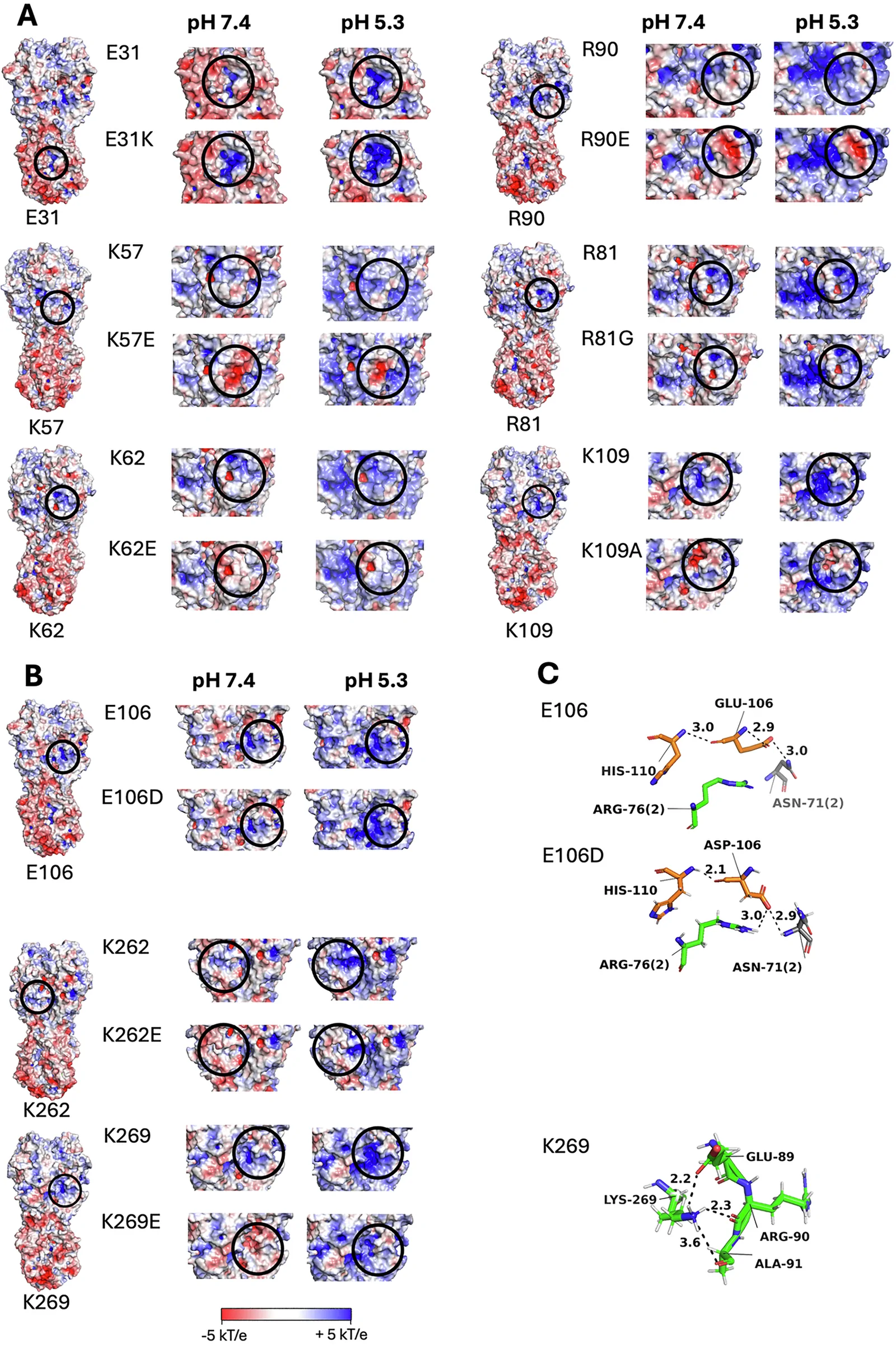
Influence of mutations on surface potential and polar interactions in HA1 subunit. **A**, **B** Surface potential at pH 7.4 and 5.3. **C** Polar interactions of mutation sites 106 and 269. Colouring of subunits for sites 106 and 269: HA1 – orange; HA2 of the same monomer – green; HA2 of the neighbouring monomer – grey.

Glu31 is located on the surface of the ECD (see Fig. 4A). The stabilising mutation results in a loss of polar interaction with Arg321, while still retaining interaction with Thr28. Since the site is in a negatively charged environment, a positively charged residue such as lysine can stabilise the prefusion conformation, particularly at an acidic pH (Fig. 4A).

#### Lys57Glu and Lys62Glu

The Lys57Glu and Lys62Glu mutations locally reduce the positive surface potential of the ECD that dominates in this region (Fig. 4A). The reduction in the strongly pronounced positive surface potential, particularly at acidic pH, caused by the mutations reduces its repulsive nature and can thus contribute to the stabilisation of the ECD. Other neutral amino acids (Lys57: Gly, Ile, Gln; Lys62: Thr, Tyr, Trp) also promote stability in this way.

#### Arg81Gly

We presume that the Arg81Gly mutation stabilises the prefusion conformation also due to an altered surface potential. The Arg81 residue is located in the HA1 head domain which has a positive potential to which the residue contributes (Fig. 4A). The mutation to glycine reduces the repulsive nature of this region, particularly at an acidic pH, which could lead to stabilisation. The stabilising effect of this mutation may have another, non-mutually exclusive cause. Adjacent to Arg81 are the highly hydrophobic residues Ile80 and Phe79. Mutations in Ile80 are always destabilising, whilst mutations in Phe79 are stabilising only if they involve neutral, hydrophobic amino acids. This shows that the hydrophobic environment is essential for the stability of the ECD and that this environment can be strengthened in its stabilising effect by appropriate mutations of Arg81 (e.g., Ile, Val, Met), which simultaneously reduce the local positive surface potential.

#### Arg90Glu

Like other mutations already mentioned, the Arg90Glu mutation reduces the local positive surface potential and thereby may also contributes to the stability of the ECD at acidic pH (Fig. 4A, see also the Lys269Glu mutation). However, with the exception of Lys, all other amino acids also increase the stability of the ECD, which can also be explained by the reduction in positive surface potential.

#### Glu106Asp

As shown on Dadonaite’s study, this mutation greatly increases the stability. The introduction of Asp maintains the existing interaction with Asn71_(2)_ as found in the wild type (wt) (Fig. 4B, C), but an additional interaction also occurs between Asp106 and Arg76_(2)_ of the neighbouring monomer. Therefore, in addition to stabilising the HA1-HA2 interface of a monomer, it also stabilises the HA1-HA2 interface between two monomers. It is noteworthy that the local surface potential is affected, even though Asp is also a negatively charged amino acid (Fig. 4B). The locally very pronounced positive potential is weakened by Asp, which could also support stabilisation at an acidic pH.

#### Lys109

This residue site is located in a short helix of the HA1 head domain (Fig. 4A). Two mutations at position 109 (Ala and Gln) stabilise the prefusion conformation at acidic pH levels^1^. This may be due to an effect on the local surface potential, whereby Lys109 contributes to the positive surface potential of this ECD region. This effect is enhanced at lower pH, which could potentially cause repulsive forces that destabilise the prefusion conformation. Mutations to Ala or Gln at position 109 reduce this effect and stabilise the structure at acidic pH. The Lys109Gln mutation is more stabilising than the Lys109Ala mutation, likely due to a polar interaction with Tyr105.

#### Lys262Ile

Dadonaite et al. (2024)^1^ found that the prefusion conformation of the Lys262Ile mutant was stable even at a pH of 5.3. Lys262 is close to other positively charged residues, such as Arg81, Lys109 and Lys269. The repulsive positive surface potential, to which Lys contributes, is enhanced at acidic pH due to the protonation of these residues (Fig. 4B). This effect is reduced by Ile, which may explain the enhanced stability at acidic pH (see also the Lys269Glu mutation). Other amino acids, such as Glu, Gln and Leu, also contribute significantly to an increased stability. They all have in common that they reduce the positive surface potential.

#### Lys269Glu

Although the mutation causes a loss of polar interaction with various residues (Glu89, Arg90 and Ala91), the locally changing surface potential could cause an increase in stability, as reported^1^ (Fig. 4B, C). The potential reduction in stability at low pH due to local repulsive forces caused by the protonation of Lys269 would be offset by the negatively charged Glu residue. As with site 262, the stability is significantly increased by amino acids such as Glu, Gln and Trp, which reduce the positive surface potential.

#### Val315-Asn104_(__2)_

There is a weak polar contact which exists between Val315 and Asn104_(2)_ and is localized within the stem region of a monomer (not shown). Mutations at Val315 generally stabilise the conformation, particularly when substituted with Arg or Asn. The Arg variant forms an additional polar bond with Gln25 of HA1, which is likely to explain the stabilisation. Although most Asn104_(2)_ mutations could not be experimentally characterised^1^, the characterised ones decreased stability, indicating that this intersubunit contact is critical for structural integrity.

#### Thr318

Thr318 is located in the stem region of HA close to the fusion peptide pocket. It forms polar interactions with His38 and His111_(2)_ (not shown). It contributes to conformational stability, as only the Ile substitution has a moderate stabilising effect on H5N1 clade 2.3.4.b HA^1^. This aligns with the findings of Imai et al. (2012)^18^, who reported a 0.3 pH unit reduction in fusion pH for HA of A/Vietnam/1203/2004 (H5N1). This effect is likely due to the higher hydrophobicity of Ile compared to Thr, which promotes interactions with residues 314–317 (Leu-Val-Leu-Ala), Gly319, Ile320, Ala44_(2)_, and Ile45_(2)_ of the adjacent HA2 subunit. Its proximity to the fusion pocket suggests that Ile may enhance embedding of the hydrophobic fusion peptide.

In summary, stabilising HA1 mutations are mainly found in the lower head domain above the stem region and act by reducing local positive surface charge or by strengthening inter- and intramolecular interactions. Substitutions such as Glu31Lys, Lys57Glu, and Arg90Glu lower electrostatic repulsion under acidic conditions, whereas mutations like Glu106Asp and Thr318Ile enhance stability through additional polar contacts or hydrophobic packing near the fusion peptide.

### Stabilising mutations in the HA2 subunit

As already mentioned, the HA2 stem and fusion peptide regions are highly conserved and critical for HA trimer stability, with most mutations being destabilising (Fig. 2 and S2). However, Dadonaite et al. (2024)^1^ identified potential stabilising mutations at selected HA2 sites (Fig. 3), primarily in the long helix and its connecting region to the short helix. The latter partially shields the lower part of the long helix from its surrounding. No stabilising mutations were found in the short helix, underscoring the importance of this region for ECD stability. We next analysed the molecular basis of mutations at the sites shown in Fig. 3 that may enhance ECD stability (see Supporting Information for Gly4_(2)_, Ala5_(2)_, Asp112_(2)_, and Asn117_(2)_).

#### Asp57_(2)_Tyr

This mutation preserves the polar interaction between Asp57_(2)_ and Asn54_(2)_, both located in the same HA2 helix (not shown), while neutralizing the slightly negative surface potential of Asp57_(2)_ at pH 7.4 and 5.3. Thr and Gly substitutions are also stabilising. Overall, neutral amino acids enhance ECD stability, whereas hydrophobic ones do not; however, the molecular basis remains unclear.

#### Arg68_(2)_Ser

**Fig. 5:**
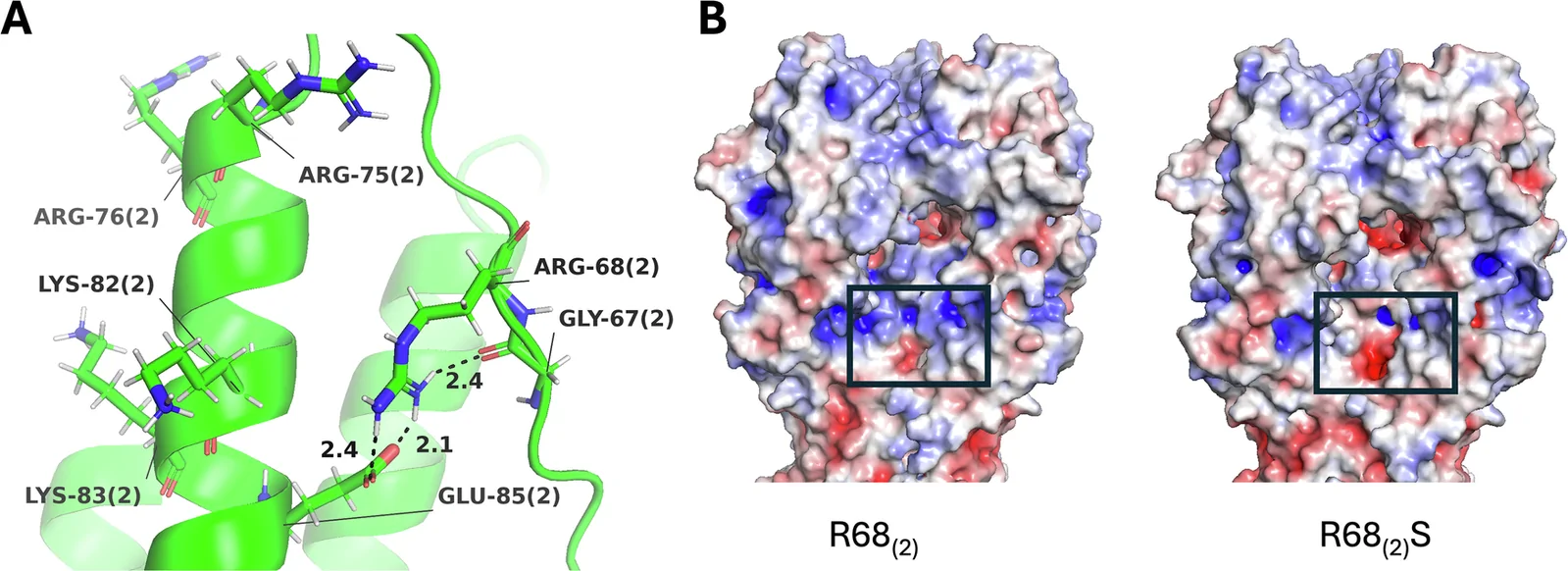
Polar interactions and surface potential effects of Arg68_(2)_ mutations. **A** Polar interactions of Arg68_(2)_ with residues within the same HA2 subunit. **B** The mutation Arg68_(2)_Ser reduces the local positive surface potential of the ECD (**B**).

Arg68_(2)_ forms polar interactions with Gly67_(2)_ and Glu85_(2)_. Glu85_(2)_ resides in the long HA2 helix, whereas Arg68_(2)_ and Gly67_(2)_ are located in the loop connecting the two HA2 helices (Fig. 5A). Nearby residues Arg75_(2)_ and Arg76_(2)_ in the loop, and Lys82_(2)_ and Lys83_(2)_ in the long helix cluster around Arg68_(2)_ (Fig. 5B; for Lys82_(2)_ and Lys83_(2)_ see Supporting Information) and contribute to a locally positive surface potential, particularly at acidic pH (Fig. 5). The Arg68_(2)_Ser mutation disrupts interactions with Gly67_(2)_ and Glu85_(2)_ without forming new ones, but reduces the local positive surface potential, which may explain its stabilising effect (Fig. 5B). Consistently, substitutions with Glu, Gln, Asn, or neutral and hydrophobic residues also enhance stability.

#### Ile77_(2)_Leu

**Fig. 6:**
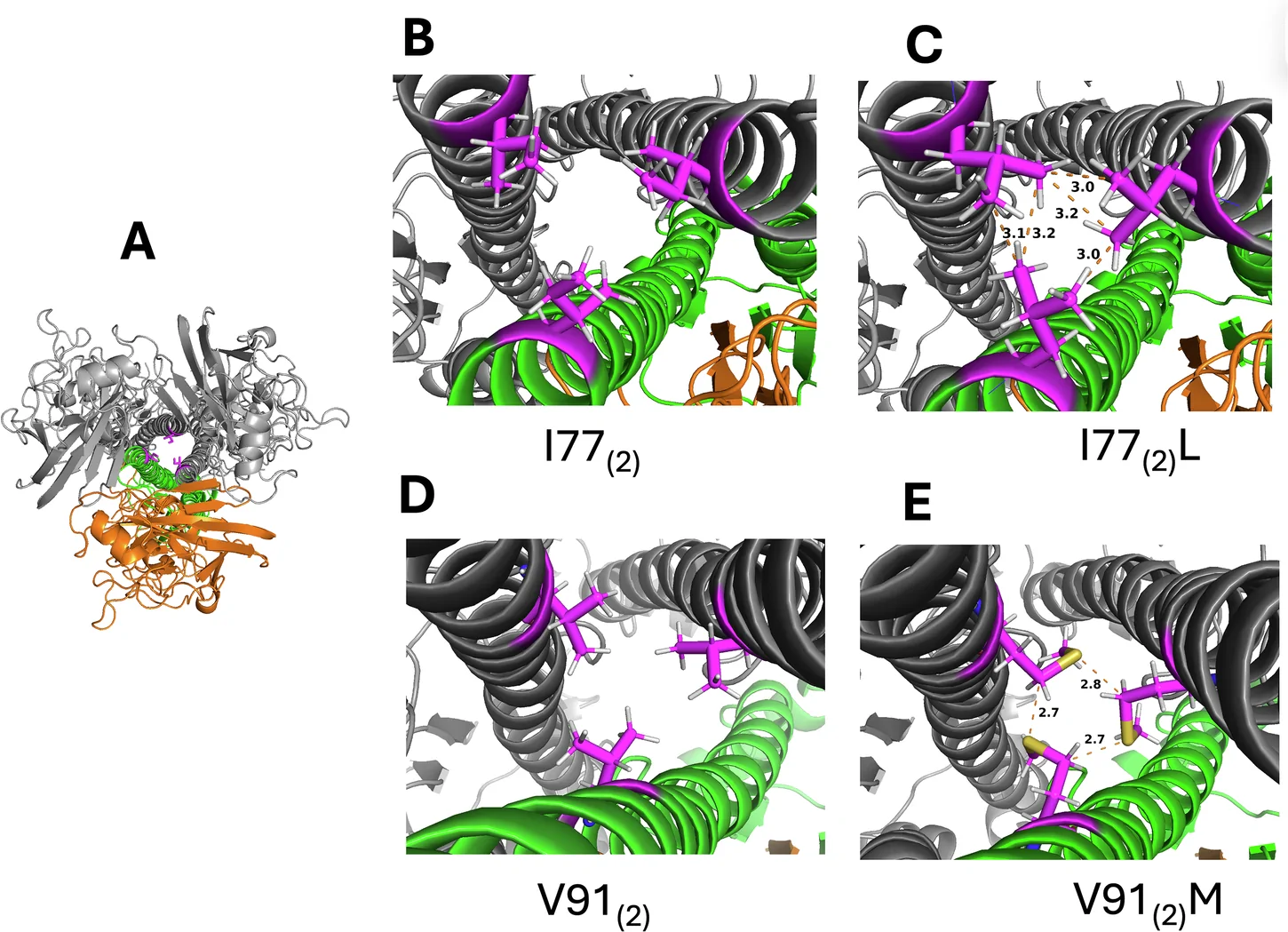
Hydrophobic interactions between long helices of HA2 subunits. **A** Top view on the trimer. The hydrophobic interactions (orange dashed lines) of the mutations Ile77_(2)_ (**B**, **C**) and of the Val91_(2)_Met (**D**, **E**) between the three long helices of the HA2 subunits are shown.

Dadonaite et al. (see Fig. 4D in ref. ^1^) found that the Ile77_(2)_Leu mutation stabilises the HA structure at an acidic pH. This site, being close to the HA head domain, is located in the long helix of the HA2 subunit. Here, the long helices of the three HA2 subunits are in close proximity to each other. Unlike Ile, Ile77_(2)_Leu of the three HA helices form several hydrophobic interactions among them, explaining the stabilising effect (Fig. 6B, C).

Interactions in the upper part of the stem region contribute to the stability of the ECD. In the wt strain H5N1, there are various residues that mediate several polar and hydrophobic interactions in this region between the long helices of the HA2 subunits of a trimer. These residues exhibit mutational intolerance with regard to the stability of the ECD (see Supporting Information, e.g. Lys82_(2)_ and Lys83_(2)_).

#### Glu78_(2)_Phe

In addition to a salt bridge with Arg75_(2)_ (3.4 Å), there are polar interactions with Glu74_(2)_, Arg75_(2)_ and Lys82_(2)_ (1.9–2.4 Å) within the same HA2 (not shown). These residues are located in the top region of the same long HA2 helix. Following the Phe mutation, however, only the polar interactions with Glu74_(2)_ and Lys82_(2)_ (2.0 and 1.9 Å, respectively) remain. The highly hydrophobic, nonpolar nature introduced by the Phe mutation suggests that the ECD can be stabilised by a hydrophobic top region of the long HA2.  This interpretation is consistent with the stabilising effect of the Ile77_(2)_Leu  mutation discussed above.

#### Leu89_(2)_Glu

There are polar interactions between Leu89_(2)_ and the residues Glu85_(2)_ and Thr93_(2)_ (not shown). The interaction with Thr93_(2)_ is strengthened by the Glu (and Asp) mutations, which create an additional, stronger interaction (not shown). This could explain why the mutation has a stabilising effect on the HA2 stem region. The mutation Glu and Asp have no effect on the surface potential at either pH 7.4 or pH 5.3.

#### Asp90_(2)_Glu

**Fig. 7:**
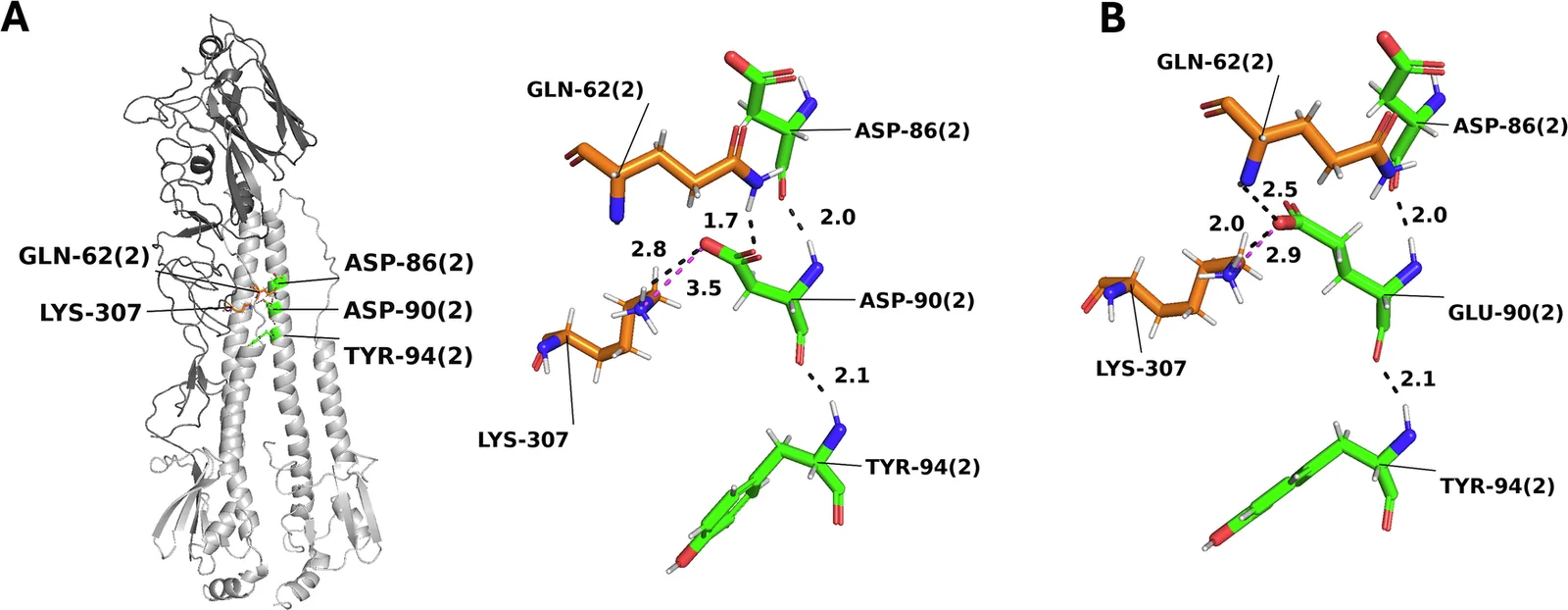
Influence of the mutation Asp90_(2)_Glu on polar interactions in the HA trimer. **A** Localization of Asp90_(2)_ and its interaction with other residues between the HA2 subunit (light grey; with relevant residues in green) and the HA1 (dark grey) and HA2 subunits (light grey) of the neighbouring monomer (with relevant HA1 and HA2 residues in orange). **B** Asp90_(2)_Glu. Salt bridges of Lys307 are shown in magenta.

Asp90_(2)_ is located in the long HA2 helix (Fig. 7A) and forms a network of polar interactions within the helix, with Gln62_(2)_ in the adjacent HA2 helix, and with Lys307 of the neighbouring HA1 subunit (Fig. 7A), including a 3.5 Å salt bridge. This network is retained in the Asp90_(2)_Glu mutation (Fig. 7B), with altered polar interactions and a strengthened interaction with Lys307 via a shorter salt bridge (2.9 Å) and additional polar contacts, stabilising the ECD.

The Asp90_(2)_Met mutation is also stabilising. Polar interactions within the same HA2 helix (with Tyr94_(2)_ and Asp86_(2)_) and a nonpolar interaction with Lys307 are maintained. Within 4 Å of position 90_(2)_, numerous nonpolar residues are present in both the same HA2 subunit (Gly, Phe, Val, Leu, Trp) and the neighbouring HA2 subunit (Met, Asn, Thr, Gln, Trp). The hydrophobic nature of Met likely promotes stabilising interactions; consistent with its tendency to be buried in protein cores, it supports packing and structural stability^61^. The surface potential is unaffected by this mutation.

#### Val91_(2)_Met

Polar interactions exist between Val91_(2)_, Gly87_(2)_ and Asn95_(2)_, which are all located on the same helix (not shown). These interactions are preserved by the Val91_(2)_Met mutation. This has a stabilising effect comparable to that of the Ile77_(2)_Leu mutation, whereby the side chains of the Met residues are oriented towards the centre of the three long HA2 helices. This leads to hydrophobic interactions between the three Met residues.

#### Asp95_(2)_Glu

**Fig. 8:**
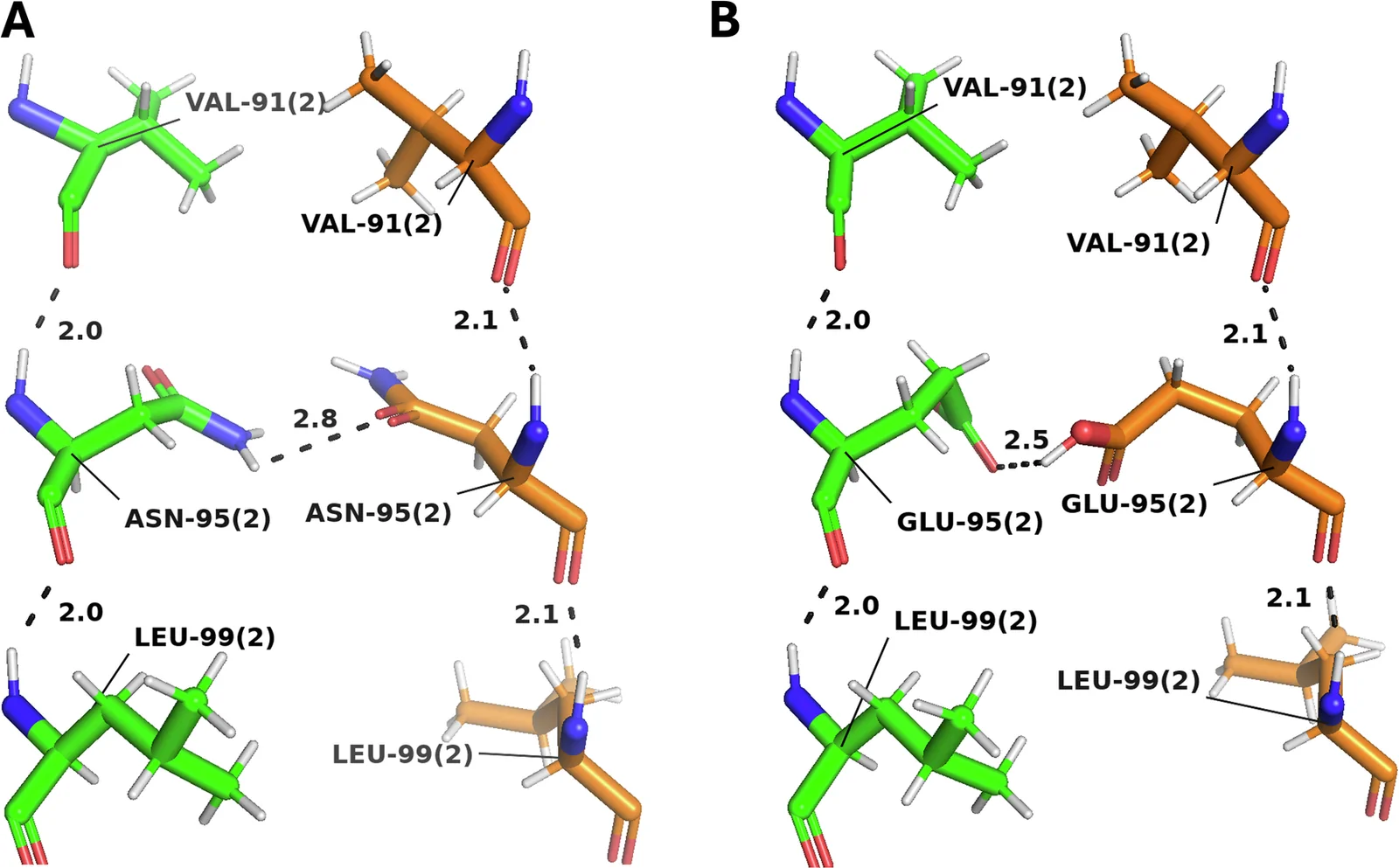
Influence of Asn95_(2)_ mutation on interaction between HA2 subunits. Interaction between two neighbouring HA2 subunits via Asn95_(2)_ (**A**) and Asn95_(2)_Glu (**B**), respectively.

Polar interactions between Asn95_(2)_ residues of adjacent long HA2 helices are maintained across pH values and are preserved in the Asp95_(2)_Glu mutation. The Glu–Glu interaction (2.5 Å) is stronger than the Asp–Asp interaction (2.8 Å) based on bond length (Fig. 8), while polar contacts with Leu99_(2)_ and Val91_(2)_ remain in both cases.

Asp95_(2)_Met is also stabilising. Methionine side chains, oriented toward the centre between the three long helices, interact via hydrophobic forces (3.0 Å). Its stabilising effect likely reflects its hydrophobic character and role in core packing, particularly given the predominantly hydrophobic surrounding residues (Val, Trp, Thr, Ala, Leu), consistent with observations for Asp90_(2)_Met. None of the mutations affects the surface potential.

#### Leu98_(2)_Met

Leu98_(2)_ forms polar interactions with Tyr94_(2)_ and Met102_(2)_ within the same HA2 helix. The Leu98_(2)_Met mutation preserves these interactions and introduces additional hydrophobic interaction with Tyr94_(2)_ and Met102_(2)_ (not shown), as well as a hydrophobic interaction with Ile55_(2)_ of the neighbouring HA2 helix, potentially enhancing ECD stability. The surface potential remains unaffected.

#### Leu99_(2)_Met

**Fig. 9:**
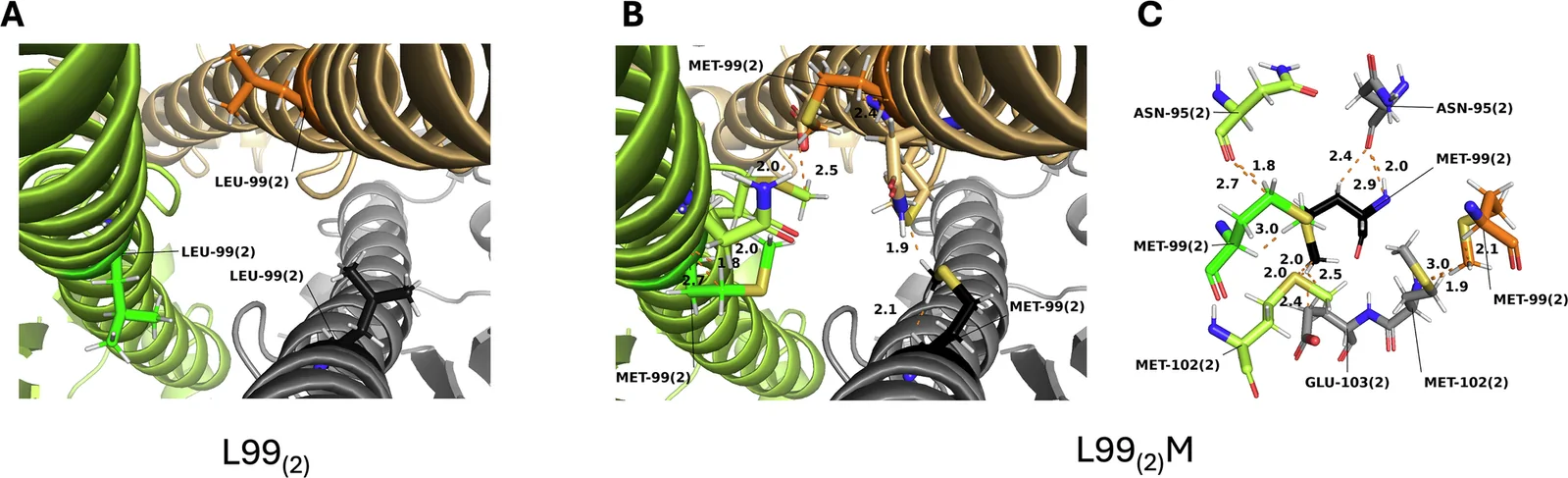
Hydrophobic interaction network formed by Leu99_(2)_Met mutation. **A** Top view on Leu99_(2)_ residues. **B, C** Mutation of Leu99_(2)_ by Met introduces a local network of hydrophobic interactions (orange) between various residues (**B**, **C**). Note that the position of the residues in (**C**) has been rotated relative to their position in (**B**) to make the various interactions more clearly visible.

Both Leu99_(2)_ (Fig. 9A) and the Leu99_(2)_Met (Fig. 9B, C) mutation form polar interactions with Asn95_(2)_ and Glu103_(2)_ on the same HA2 helix (not shown). However, unlike Leu, Met forms a network of hydrophobic interactions due to its orientation towards the centre between the three long HA2 helices (Fig. 9).

#### Arg106_(2)_Glu and Arg106_(2)_Lys

**Fig. 10:**
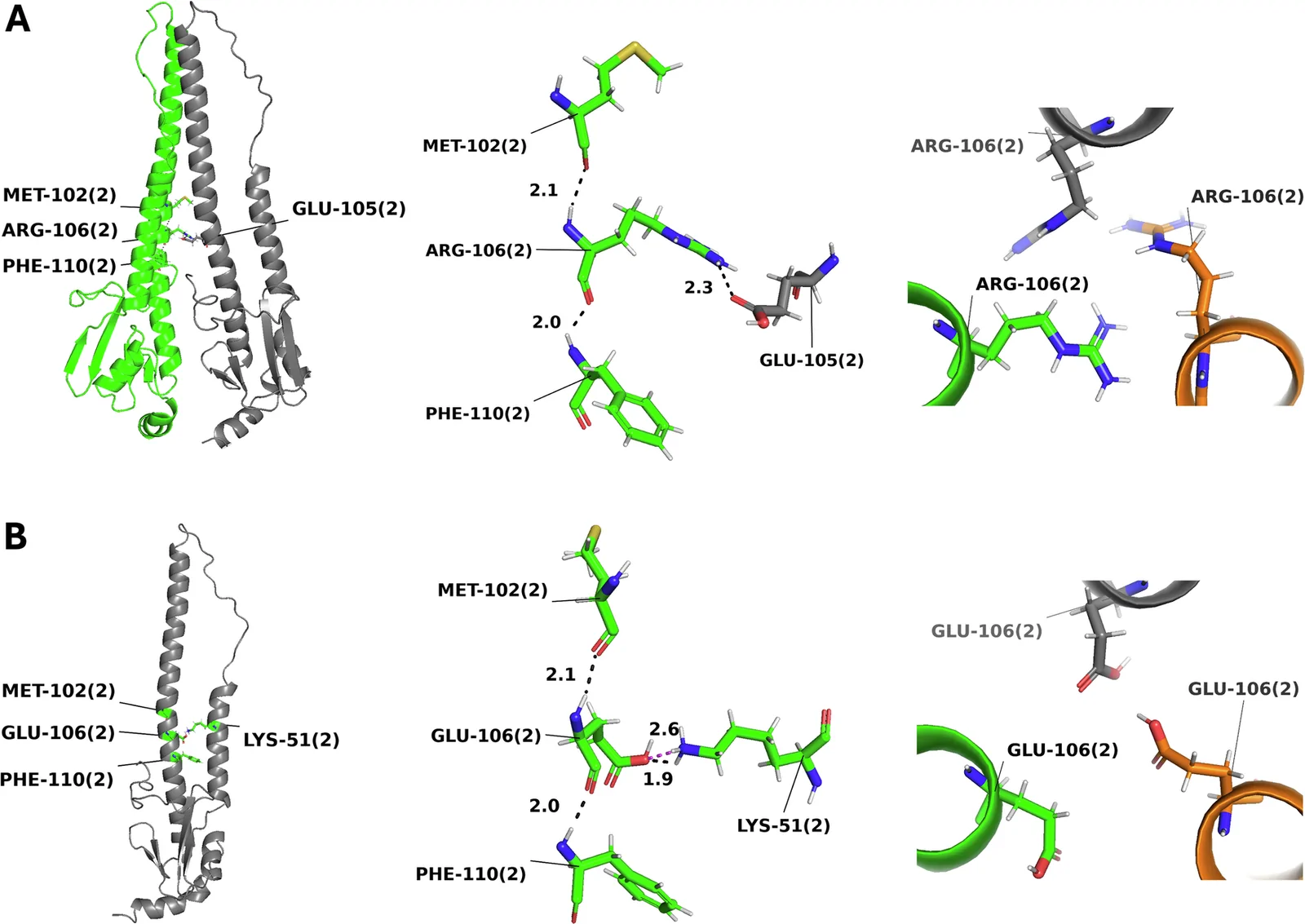
Consequences of Arg106_(2)_ mutation on inter- and intra-helical interactions in HA2. **A** Interaction between two neighbouring HA2 subunits via Arg106_(2)_ und Glu105_(2)_. **B** Interaction between the long and short helix of a HA2 subunit via Arg106_(2)_Glu and Lys51_(2)_. In the right panel the orientation of the residues Arg106_(2)_ (**A**) and Arg106_(2)_ (**B**) of the three long HA2 helices are shown. Salt bridge (**B**) is shown in magenta.

Arg106_(2)_ forms polar interactions within the same helix and with Glu105_(2)_ of a neighbouring HA2 subunit (Fig. 10A); the latter interaction is lost in the Arg106_(2)_Lys and Asp106_(2)_Glu mutations. In Asp106_(2)_Glu, a new interaction with Lys51_(2)_ in the short HA2 helix of the same subunit is formed and stabilised by a 2.6 Å salt bridge (Fig. 10B). The stabilising effect may also result from reduced repulsion - especially at acidic pH - between Arg106_(2)_ side chains projecting into the stem centre, an effect diminished by Arg106_(2)_Lys and more strongly by Arg106_(2)_Glu. Consistent with Dadonaite et al. (2024), only charged substitutions appear to confer stability, whereas neutral or nonpolar residues do not.

#### Asn117_(2)_Glu

**Fig. 11:**
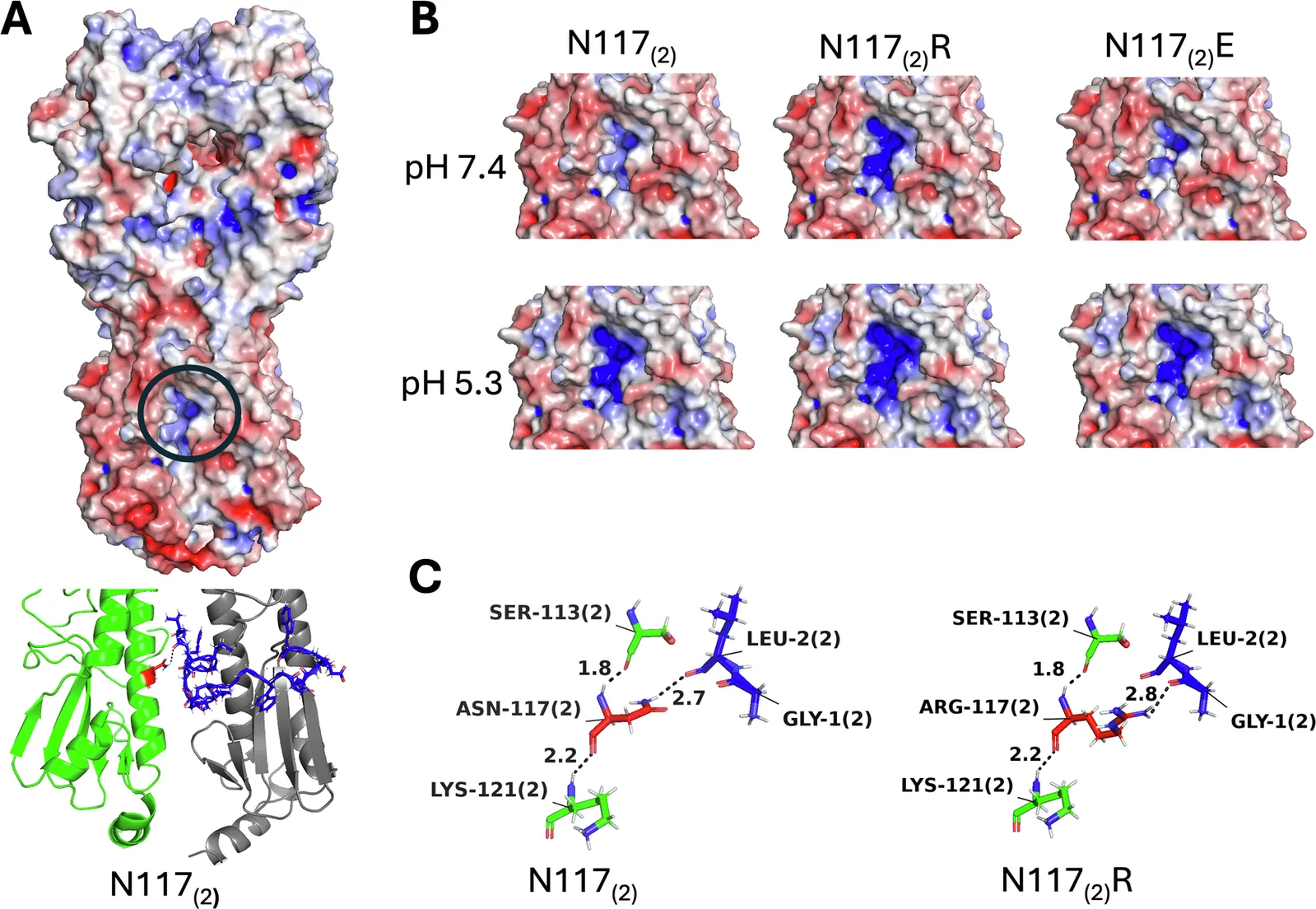
Influence of mutation of Asn117_(2)_ on the surface potential. **A**, **B** Polar interactions of Asn117_(2)_ localised in the lower part of the stem region and the influence of the mutation Asn177_(2)_Glu on the surface potential of the ECD at pH 7.4 and 5.3. **C** Polar interactions of Asn117_(2)_ and Asn117_(2)_Arg. Colouring of subunits: HA2 of the same monomer – green, relevant residue - red; HA2 of the neighbouring monomer – grey, relevant residues - blue.

This residue is located in the helical region of HA2, close to the viral envelope. Dadonaite et al. (2024)^1^ found that this mutation stabilises the HA structure at acidic pH (see Fig. 4D in ref. ^1^). Asn117_(2)_ forms an intrasubunit interactions with Ser113_(2)_ and Lys121_(2)_, as well as an intermonomeric contact with Leu2_(2)_ of the neighbouring HA2 subunit. Leu2_(2)_ marks the N-terminus of the fusion peptide. The Asn117_(2)_Glu mutation leads to a loss of the interaction with Leu2_(2)_. Additionally, this mutation causes a considerable shift in the local surface potential from slightly positive to negative values (Fig. 11). Therefore, Asn117_(2)_Glu could reduce the repulsive nature of the region through protonation at low pH, thereby stabilising the prefusion conformation. However, it should be noted that other mutations also stabilise the prefusion conformation. These include neutral amino acids that provide a more hydrophobic nature matching the property of the fusion peptide.

In summary, potentially stabilising HA2 mutations occur mainly in the long helix and the connecting loop. They often increase stability by improving interhelical packing through hydrophobic substitutions, strengthening polar networks, or reducing electrostatic repulsion in charged regions. Methionine substitutions are especially notable because they support hydrophobic core packing within the helix bundle.

### Histidine residues as pH sensors for the HA conformational change

Several studies have identified specific histidine residues as key pH sensors that can trigger the HA conformational change upon protonation. Histidine is uniquely suited to this role as its protonation state changes within the physiological pH range (pH 7–6). At neutral pH, histidine is deprotonated and uncharged whereas at acidic pH, near its pKa (~6), it becomes protonated. However, the effective pKa of a histidine side chain depends on its local environment^62,63^.

**Fig. 12:**
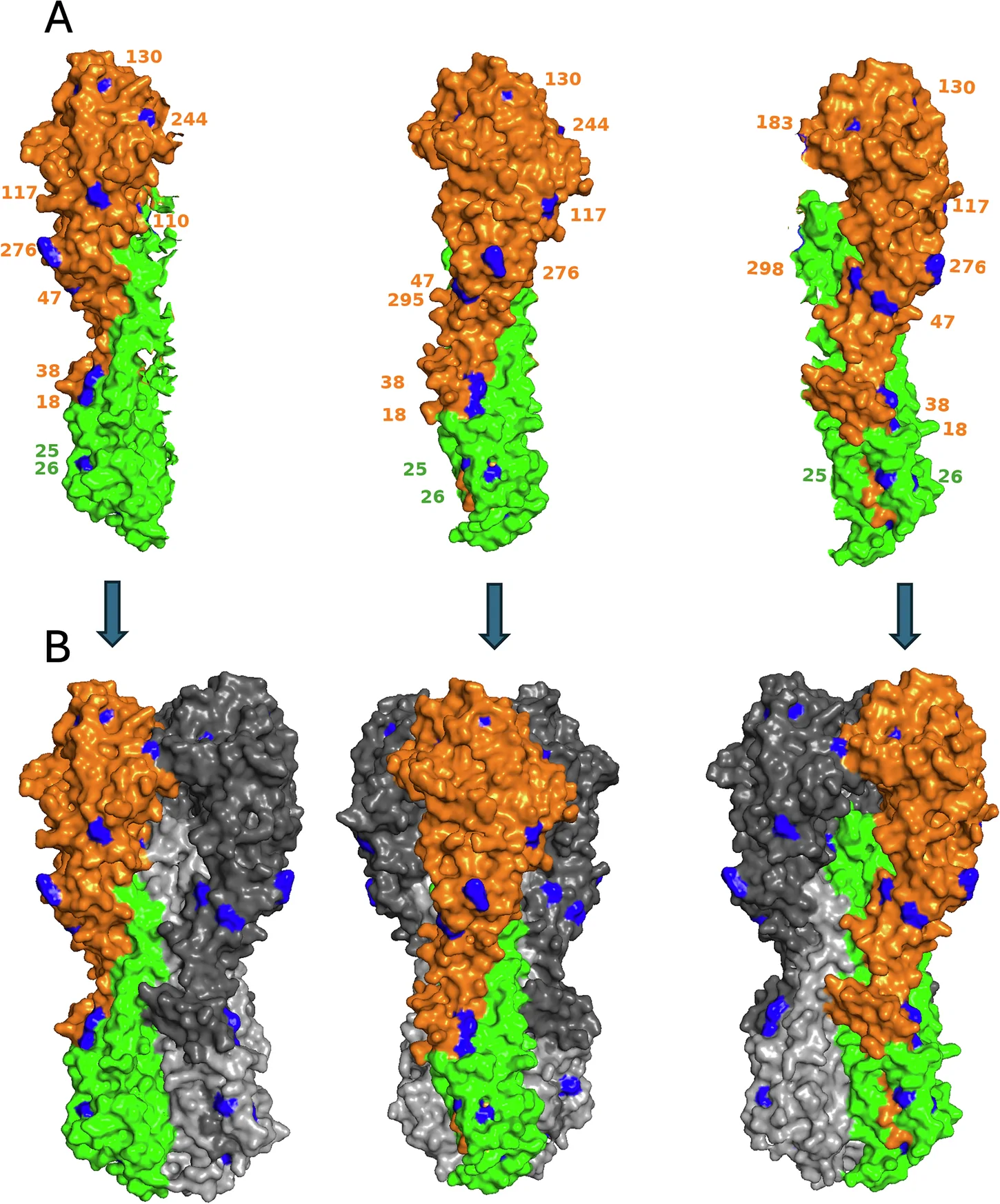
Localisation of surface exposed histidine residues in the ECD of HA. **A** The His residues are shown (in blue) only for one monomer in three different positions. **B** Position of the monomer shown in panel A within the trimer.

In total, there are 16 histidine residues in the ECD of the HA monomer of A/Texas/37/2024, four of which are located in the HA2 subunit. All residues except His111_(2)_, are localised on the surface of the ECD, albeit exposed to varying degrees (Fig. 12). Some histidine residues that are either conserved within a single subtype or across several subtypes have been suggested as possible triggers of the pH-dependent conformational change of HA. These include His18 and His184 in HA1, and His111_(2)_ and His142_(2)_ in HA2 of A/Texas/37/2024^12,62,64–66^ (Figure S8). With a few exceptions, mutations of histidine residues negatively affect HA stability and/or viral entry (Dadonaite et al. (2024))^1^, for example mutation of His111_2_ (Figure S9). Exceptions include HA1 histidine residues 18, 38, and 110. Substituting these positions with non-charged residues (e.g., Ser or Thr), or with a positively charged amino acid in the case of His38, have been shown to stabilise HA.

#### His18

His18 is in close proximity to the fusion peptide (Fig. S6) and forms a polar contact with Trp21_(2)_ (not shown). Protonation of His18 can destabilise this region and promote peptide release during the conformational change. Substituting His18 with Gln has been shown to lower the fusion pH threshold by ~0.3 units in H5 HA^9,67–69^. Dadonaite et al. (2024)^1^ confirmed this stabilising effect, as Gln lacks protonation and forms an additional polar contact with Gly20_(2)_ (not shown), thereby enhancing local stability at low pH. Similarly, His18Phe exerts a stabilising influence on the ECD.

#### His110

This exposed residue in the HA1 head, forms polar contacts with Glu106 and Ser113 and interacts with HA2 via Glu69_(2)_ (not shown). Dadonaite et al. (2024)^1^ found that substitutions with neutral residues enhance ECD stability, which is consistent with reports that substitution of His110 with Tyr lowers the fusion pH from 5.6 to 5.2 and increases airborne transmission in ferrets^37,47^. This effect likely reflects reduced electrostatic repulsion, as His110 is surrounded by positively charged residues (Lys109, Arg114, Lys262, Arg75_(2)_ and Arg76_(2)_). Protonation at low pH would otherwise destabilise the structure. Tyrosine substitution mitigates this effect stabilising the prefusion conformation. However, Mair et al. (2014) ^62^ noted that the conservation of His110 in only a few subtypes (H2, H5, H13, H16) suggests that it is not the main trigger of the conformational change.

## Discussion

In this study, we examined how individual mutations in the ECD of HA of the currently circulating H5N1 clade 2.3.4.4b virus affect interactions at specific sites, using deep mutational scanning data from Dadonaite et al. (2024)^1^. In contrast to Dadonaite et al. (2024), who identified stabilising mutations experimentally, our study provides a structural interpretation of how these substitutions alter local interaction networks and surface electrostatics in the ECD. This analysis is valuable for monitoring critical mutations and providing initial insights into viral adaptation to human host cells. Overall, our structural and mutational analyses indicate that H5N1 clade 2.3.4.4b HA possesses a substantial capacity to accommodate stabilising substitutions that enhance resistance to acid-induced conformational change. The identified mutations act through complementary mechanisms, which include the neutralisation of surface-exposed acidic residues, the strengthening of hydrogen-bonding networks, and the reinforcement of trimeric interfaces. These mechanisms are intended to preserve the metastable prefusion architecture at lower pH^12,13,15,17^. Such adaptations are analogous to the evolutionary trajectory observed in human-adapted influenza subtypes, where increased HA acid stability preserves the metastable HA conformation along the airborne transmission via respiratory droplets and the passage of the mildly acidic environment of the upper respiratory tract. Consequently, the emergence of these stabilising mutations in circulating avian strains could signify progressive adaptation toward mammalian hosts. Therefore, continuous genomic surveillance and analysis of the structural consequences of mutations of these variants are essential to assess their potential impact on viral fitness, transmissibility, and pandemic risk. However, one has to keep in mind that the effects of mutations on acid stability do not necessarily correlate strongly with their effects on cell entry^1,70^, indicating that these phenotypes should be considered separately.

To place these mutational effects into a structural context, we analysed the residue interaction networks within the ECD of wt H5N1. The analysis of residues in the ECD of wt H5N1, for which most mutations destabilise the ECD (see Supplemental Information), revealed that they are embedded in local networks of polar interactions spanning HA1 and HA2, with the stem region contributing strongly to structural integrity. In the upper stem, residues such as Lys51_(2)_, Asn114_(2)_, and the Lys82_(2)_-Lys83_(2)_-Asn81_(2)_ cluster act as stabilising nodes by forming intra- and interhelical contacts that link HA2 helices and connect them to HA1. Interactions in the lower region of the long HA2 helix likewise support trimer integrity, as mutations in these residues are consistently detrimental. The fusion peptide region is embedded in a dense network of short-range polar interactions, reflecting its dual role in maintaining prefusion stability and enabling membrane fusion. In the HA1 head domain, networks including His184 contribute to stability, with its protonation weakening polar contacts and supporting its role as a pH sensor for conformational change. These structural features provide a mechanistic basis for the stabilising effects of mutations described above, particularly those that modulate electrostatic interactions and reinforce interdomain contacts.

**Fig. 13:**
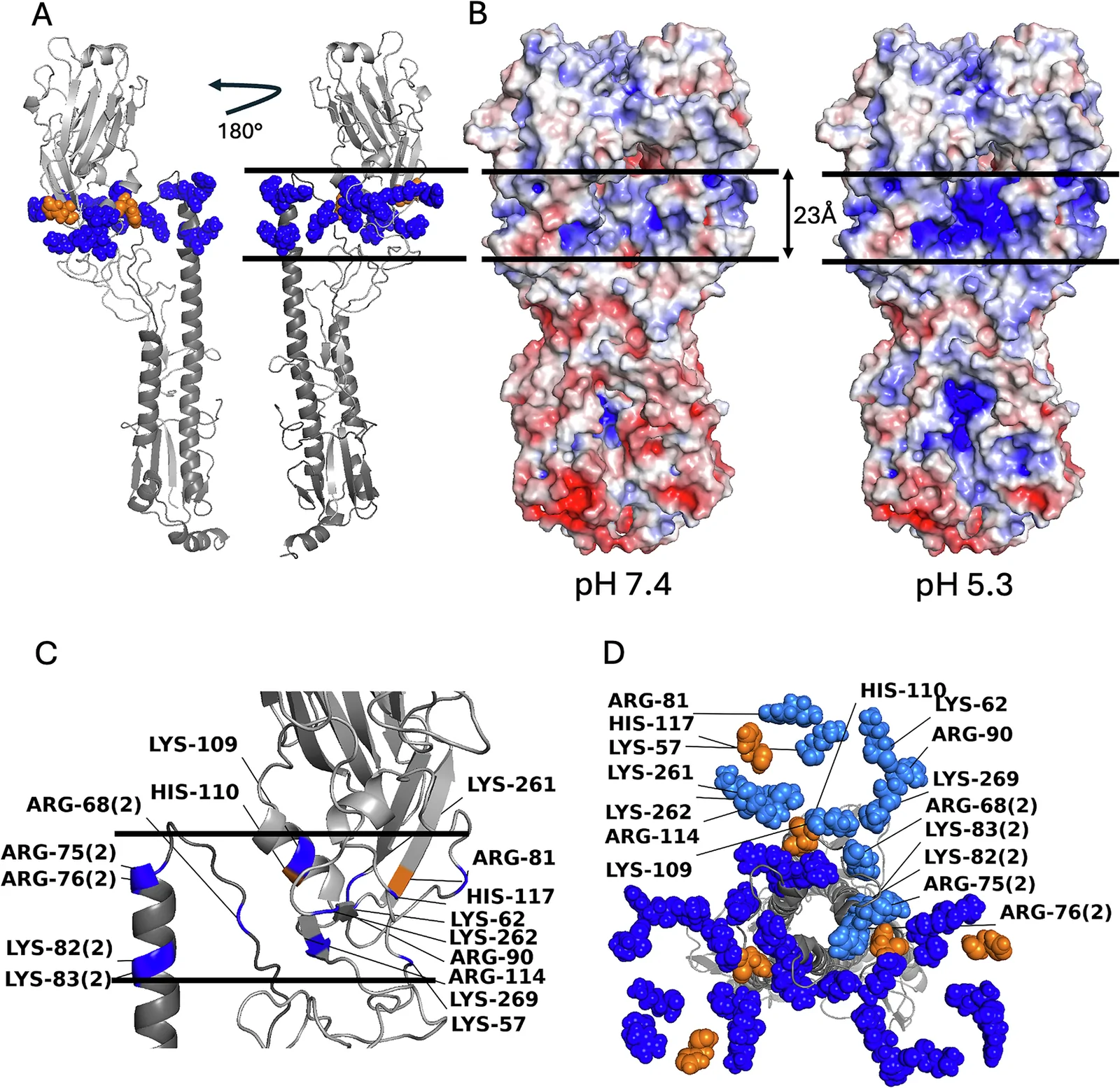
Localisation and impact of positively charged residues in the lower HA1 head domain on surface potential. Localisation of amino acids in the lower region of the wt HA1 head domain (**A**), which induce a pronounced local positive surface potential at neutral and, in particular, acidic pH (**B**). These residues cover the entire cross-section of a monomer (**C**, **D**). By introducing appropriate mutations in some of these residues, which lead to a reduction in the positive surface potential, the prefusion conformation of the ECD can be stabilised (for details, see text).

**Fig. 14:**
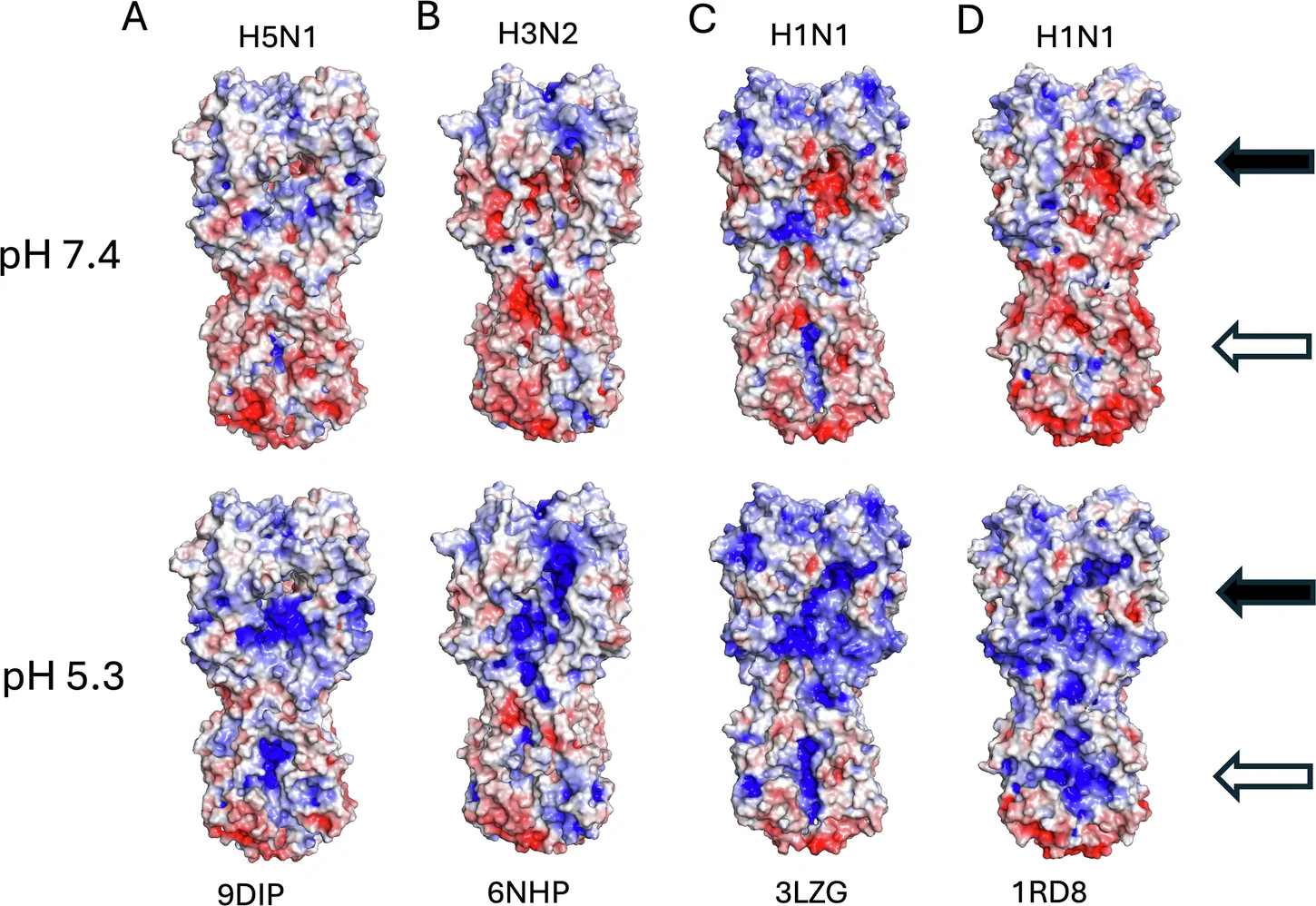
Comparison of the surface potential of strains of different IAV subtypes at neutral pH (7.4) and acidic pH (5.3). **A** A/Texas/37/2024 (H5N1); (**B**) A/Hong Kong/1/1968 (H3N2); (**C**) A/California/04/2009 (H1N1); (**D**) A/South Carolina/1/1918 (H1N1). The arrows indicate regions of pronounced positive surface potential, particularly at pH 5.3. The pdb codes are given.

Potential stabilising HA1 mutations are mainly located in the lower head domain above the stem region. Here positively charged amino acids are densely organised in a small region of the lower wt head domain, spanning the full monomer cross-section, and the repulsive potential may contribute to triggering conformational change (Fig. 13). Mutations likely stabilise HA by reducing the pronounced positive surface potential, which may lower electrostatic repulsion at acidic pH and help to maintain the prefusion conformation. We note that changes in local surface potential are likely one contributor to ECD stability, but not a quantitative predictor, as local stability also depends on additional interactions not captured by this parameter. Further work is needed to determine whether this mechanism is conserved across IAV subtypes. Surface-potential comparisons of H5N1, H3N2, and H1N1 strains show similarly positive regions at acidic pH, although their organization differs (Fig. 14), which does not exclude a role in conformational conversion.

Based on recent studies by Bloom and colleagues, the organisation of stabilising and destabilising interactions within the ECD appears to be only weakly conserved across subtypes. In line with the H5N1 analysis by Dadonaite et al. (2024)^1^, comparable assessments of HA stability have also been performed for human-adapted H3N2 (A/Massachusetts/18/2022) and H7N9 (A/Anhui/1/2013) strains (Yu et al., 2026^70^; Ahn et al., 2026^71^). Despite sharing highly conserved structures and cell entry functions, HA subtypes H3, H5 and H7 exhibit significant sequence divergence, with only 40–47% amino acid identity^71^. Deep mutational scanning revealed that approximately half of the HA sites exhibit significantly different amino acid preferences across these subtypes. While the analysis confirmed that the HA2 stem domain is more conserved and the HA1 head and receptor-binding regions show greater variation in response to immunological and host-related pressures, it also demonstrated that proteins with highly similar structures can evolve dramatically different site-specific evolutionary constraints. This limits the transferability of mutational data between subtypes and highlights the importance of subtype-specific information in vaccine design and viral surveillance.

However, the present approach considers each mutation in isolation and does not account for multiple simultaneous mutations. Xie et al. (2025)^25^ highlighted the challenges of analysing the synergistic effects on transmissibility and virulence, given the limited methodological and functional study options due to ethical and safety constraints. Dosey et al. (2025)^72^ demonstrated that simultaneous mutations in the HA stem of H5N1 clade 2.3.4.4b can stabilise the protein in ways not observed when mutations are studied individually. The approach employed in this study has the potential to characterise the influence of mutations occurring at multiple sites concurrently. For instance, the impact on the surface potential of the ECD would be more significant if multiple stabilising mutations characterised in this study occurred together in HA1. However, it remains unclear whether this would cause local structural changes, or whether it would have a stabilising effect. It is evident that experimental investigations would be necessary to determine this.

This approach is also relevant to understanding epistasis^73,74^, whereby compensatory mutations offset the fitness costs of otherwise detrimental escape mutations. Host adaptation and antibody evasion may drive mutations in HA, which compromise its stability and reduce viral fitness^75,76^. For example, the stem is highly conserved, making it a promising target for broadly neutralizing antibodies^77,78^. However, escape mutations in this region can disrupt the delicate balance between stability and fusogenic function^79^. Lee et al. (2023)^80^ demonstrated that escape mutations in the HA stem of pandemic H1N1 viruses incur fitness costs. However, these have been preceded by a compensatory mutation in the head domain that enhanced receptor affinity, offsetting the deleterious effects of stem alterations^81,82^. Finally, now it can be detected in currently globally circulating pandemic IAVs of the H1N1 lineage. Similar interactions have been observed in H3N2 viruses, where co-evolving HA1 head mutations increase binding avidity and thermal stability despite the destabilising effects of individual mutations^83^.

Evaluating HA in isolation also neglects the role of the homotetrameric spike protein neuraminidase (NA). Although NA is less abundant than HA, it is essential for mucus penetration and for viral release from the host cell^84,85^. NA cleavage of sialic acids facilitates mucus traversal and host cell access^86–88^, and its activity must remain balanced with HA binding to optimize viral attachment and release^86,87,89–92^. Liu et al. (2022)^93^ showed that studying HA drift alone ignores the HA–NA balance crucial for viral fitness, as interactions between the two proteins shape antigenic evolution. Ilyushina et al. (2012)^94^ found that avian H5N1 viruses adapted to human bronchial cells via HA mutations only when NA activity was suppressed, indicating that reduced NA facilitates adaptation. Liu et al. (2022)^93^ emphasised that ignoring HA–NA interactions overlooks key drivers of antigenic evolution. Blumenkrantz et al. linked limited mammalian transmissibility of H5N1 to short NA stalks impairing mucus penetration^69^, whereas Hermann and Krammer (2025)^95^ reported that most clade 2.3.4.4b H5N1 isolates, including those in dairy cattle, retain long NA stalks. Similar HA–NA co-adaptations occur in H7N3 viruses, where HA mutations lowering activation pH coincide with NA stalk deletions reducing activity^96^.

## Conclusion

This study provides a structural interpretation of mutations that enhance acid stability of the hemagglutinin ectodomain of H5N1 clade 2.3.4.4b. Integrating deep mutational scanning with structural analysis shows that increased stability at acidic pH arises from complementary mechanisms, including modulation of surface electrostatics, reinforcement of hydrogen-bond networks, and stabilisation of intra- and intermonomeric interfaces, thereby preserving the metastable prefusion conformation under conditions relevant for mammalian infection and airborne transmission.

A key finding is that many effective stabilising mutations are located in the HA1 head domain rather than the conserved HA2 stem. These substitutions mainly reduce protonation-induced increases in positive surface potential at mildly acidic pH that would otherwise destabilise the prefusion conformation. Representative examples include Glu31Lys, Arg81Gly, Lys109Ala/Gln, Lys262Ile and Lys269Glu, underscoring the importance of electrostatic tuning for HA acid stability. Stabilising mutations can be qualitatively ranked by structural impact. Highly stabilising mutations (e.g. Glu106Asp, Lys262Ile, Lys269Glu and His110Tyr) strongly reduce electrostatic repulsion or reinforce key interfaces. Moderately stabilising mutations, such as Thr318Ile and Ile77_(2)_Leu, enhance hydrophobic packing or trimeric stem interactions, while context-dependent mutations (e.g. Asn117_(2)_Glu and His18Gln) fine-tune local pH sensitivity near the fusion peptide and likely act synergistically. Overall, the stability of HA acid is not governed by a single mutation but can be incrementally enhanced through multiple pathways. Although this indicates that there is potential evolutionary plasticity in clade 2.3.4.4b viruses, this plasticity is likely constrained by other pleiotropic effects. However, this study considers mutations individually and does not capture epistatic effects or constraints imposed by receptor binding, neuraminidase activity and polymerase function. Therefore, experimental studies combining mutations and analysing HA–NA co-adaptation will be essential. Our analysis provides a structural framework for interpreting acid-stability-associated HA mutations, the proposed mechanisms remain computationally derived hypotheses and will require direct experimental validation using purified mutant proteins, biophysical stability measurements, and ideally mutant HA structures.

In summary, this study has identified potential structural determinants of HA acid stability in the current H5N1 clade 2.3.4.4b viruses, providing a basis for prioritising mutations in genomic surveillance and evaluating their impact on pandemic risk.

## Supplementary information

Supplementary Information

## Data Availability

All data supporting the findings of this study are available within the paper and its Supplementary Information. Data are available from the corresponding authors upon reasonable request.

## References

[1] Dadonaite, B. et al. Deep mutational scanning of H5 hemagglutinin to inform influenza virus surveillance. *PLOS Biol.* **22**, e3002916 10.1371/journal.pbio.3002916 (2024). PMC11584116 39531474

[2] Drake, J. M. Understanding avian influenza mortality. *Science* **389**, 1292–1294 10.1126/science.adx7159 (2025). 40997186

[3] Fereidouni, S. et al. Genetic characterization of a new candidate hemagglutinin subtype of influenza A viruses. *Emerg. Microbes Infect.* **12**, 2225645 10.1080/22221751.2023.2225645 (2023). PMC10308872 37335000

[4] Klenk, H.-D. & Garten, W. Host cell proteases controlling virus pathogenicity. *Trends Microbiol.* **2**, 39–43 10.1016/0966-842X(94)90123-6 (1994). 8162439

[5] Stieneke-Gröber, A. et al. Influenza virus hemagglutinin with multibasic cleavage site is activated by furin, a subtilisin-like endoprotease. *Embo j.* **11**, 2407–2414 10.1002/j.1460-2075.1992.tb05305.x (1992). PMC556715 1628614

[6] Huang, R. T. C., Wahn, K., Klenk, H. D. & Rott, R. Fusion between cell membrane and liposomes containing the glycoproteins of influenza virus. *Virology* **104**, 294–302 10.1016/0042-6822(80)90334-7 (1980). 7395107

[7] Maeda, T. & Ohnishi, S. -i Activation of influenza virus by acidic media causes hemolysis and fusion of erythrocytes. *FEBS Lett.* **122**, 283–287 10.1016/0014-5793(80)80457-1 (1980). 7202720

[8] White, J., Matlin, K. & Helenius, A. Cell fusion by Semliki Forest, influenza, and vesicular stomatitis viruses. *J. Cell Biol.* **89**, 674–679 10.1083/jcb.89.3.674 (1981). PMC2111813 6265470

[9] Reed, M. L. et al. The pH of Activation of the Hemagglutinin Protein Regulates H5N1 Influenza Virus Pathogenicity and Transmissibility in Ducks. *J. Virol.* **84**, 1527–1535 10.1128/jvi.02069-09 (2010). PMC2812356 19923184

[10] Brown, J. D., Goekjian, G., Poulson, R., Valeika, S. & Stallknecht, D. E. Avian influenza virus in water: Infectivity is dependent on pH, salinity and temperature. *Vet. Microbiol.* **136**, 20–26 10.1016/j.vetmic.2008.10.027 (2009). 19081209

[11] Wit, E. D. et al. Molecular determinants of adaptation of highly pathogenic avian influenza H7N7 viruses to efficient replication in the human host. *J. Virol.* **84**, 1597–1606 10.1128/jvi.01783-09 (2010). PMC2812334 19939933

[12] Mair, C. M., Ludwig, K., Herrmann, A. & Sieben, C. Receptor binding and pH stability — How influenza A virus hemagglutinin affects host-specific virus infection. *Biochimica et. Biophysica Acta (BBA) - Biomembranes* **1838**, 1153–1168 10.1016/j.bbamem.2013.10.004 (2014). 24161712

[13] Russier, M. et al. Molecular requirements for a pandemic influenza virus: An acid-stable hemagglutinin protein. *Proc. Natl. Acad. Sci.* **113**, 1636–1641 10.1073/pnas.1524384113 (2016). PMC4760800 26811446

[14] Galloway, S. E., Reed, M. L., Russell, C. J. & Steinhauer, D. A. Influenza HA Subtypes demonstrate divergent phenotypes for cleavage activation and pH of fusion: implications for host range and adaptation. *PLOS Pathog.* **9**, e1003151 10.1371/journal.ppat.1003151 (2013). PMC3573126 23459660

[15] Russell, C. J. Hemagglutinin Stability and Its Impact on Influenza A Virus Infectivity, Pathogenicity, and Transmissibility in Avians, Mice, Swine, Seals, Ferrets, and Humans. *Viruses* **13**, 746 (2021). 10.3390/v13050746 PMC8145662 33923198

[16] Hu, M. et al. Hemagglutinin destabilization in H3N2 vaccine reference viruses skews antigenicity and prevents airborne transmission in ferrets. *Sci. Adv.* **9**, eadf5182 10.1126/sciadv.adf5182 (2023). PMC10058244 36989367

[17] Tosheva, I. I. et al. Hemagglutinin stability as a key determinant of influenza A virus transmission via air. *Curr. Opin. Virol.* **61**, 101335 10.1016/j.coviro.2023.101335 (2023). 37307646

[18] Imai, M. et al. Experimental adaptation of an influenza H5 HA confers respiratory droplet transmission to a reassortant H5 HA/H1N1 virus in ferrets. *Nature* **486**, 420–428 10.1038/nature10831 (2012). PMC3388103 22722205

[19] Richard, M. et al. Mutations driving airborne transmission of A/H5N1 virus in mammals cause substantial attenuation in chickens only when combined. *Sci. Rep.* **7**, 7187 10.1038/s41598-017-07000-6 (2017). PMC5543172 28775271

[20] Russell, C. J., Hu, M. & Okda, F. A. Influenza Hemagglutinin Protein Stability, Activation, and Pandemic Risk. *Trends Microbiol.* **26**, 841–853 10.1016/j.tim.2018.03.005 (2018). PMC6150828 29681430

[21] Pye, H. O. T. et al. The acidity of atmospheric particles and clouds. *Atmos. Chem. Phys.* **20**, 4809–4888 10.5194/acp-20-4809-2020 (2020). PMC7791434 33424953

[22] Xie, R. et al. The episodic resurgence of highly pathogenic avian influenza H5 virus. *Nature* **622**, 810–817 10.1038/s41586-023-06631-2 (2023). 37853121

[23] Verhagen, J. H., Fouchier, R. A. M. & Lewis, N. Highly pathogenic avian influenza viruses at the wild–domestic bird interface in Europe: future directions for research and surveillance. *Viruses* **13**, 212 (2021). 10.3390/v13020212 PMC7912471 33573231

[24] Peacock, T. P. et al. The global H5N1 influenza panzootic in mammals. *Nature* **637**, 304–313 10.1038/s41586-024-08054-z (2025). 39317240

[25] Xie, Z. et al. Clade 2.3.4.4b highly pathogenic avian influenza H5N1 viruses: knowns, unknowns, and challenges. *J. Virol.* **99**, e00424–00425 10.1128/jvi.00424-25 (2025). PMC12172449 40340397

[26] Nguyen, T.-Q. et al. Emergence and interstate spread of highly pathogenic avian influenza A(H5N1) in dairy cattle in the United States. *Science* **388**, eadq0900 10.1126/science.adq0900 (2025). 40273240

[27] Elsmo, E. et al. Highly Pathogenic Avian Influenza A(H5N1) Virus Clade 2.3.4.4b Infections in Wild Terrestrial Mammals, United States, 2022. *Emerg. Infect. Dis. J.* **29**, 2451 10.3201/eid2912.230464 (2023). PMC10683806 37987580

[28] Caserta, L. C. et al. Spillover of highly pathogenic avian influenza H5N1 virus to dairy cattle. *Nature* **634**, 669–676 10.1038/s41586-024-07849-4 (2024). PMC11485258 39053575

[29] Dholakia, V. et al. Polymerase mutations underlie early adaptation of H5N1 influenza virus to dairy cattle and other mammals. *bioRxiv*, 2025.2001.2006.631435 10.1101/2025.01.06.631435. PMC12905184 41545362

[30] Guo, Z. et al. Ciliated cells promote high infectious potential of influenza A virus through the efficient intracellular activation of hemagglutinin. *J. Virol.* **99**, e00685–00625 10.1128/jvi.00685-25 (2025). PMC12456007 40882005

[31] Mellace, M., Ceniti, C., Borrelli, L. & Tilocca, B. Viral protein mutations enabling mammalian adaptation in Avian Influenza A viruses: strategies for zoonotic risk mitigation and future perspectives. *Virology* **614**, 110731 10.1016/j.virol.2025.110731 (2026). 41176952

[32] Chopra, P. et al. Receptor binding specificity of a Bovine A(H5N1) Influenza Virus. *bioRxiv* 10.1101/2024.07.30.605893 (2024).

[33] Good, M. R. et al. A single mutation in dairy cow-associated H5N1 viruses increases receptor binding breadth. *Nat. Commun.* **15**, 10768 10.1038/s41467-024-54934-3 (2024). PMC11685663 39737954

[34] Song, H. et al. Receptor binding, structure, and tissue tropism of cattle-infecting H5N1 avian influenza virus hemagglutinin. *Cell*10.1016/j.cell.2025.01.019 (2025). 39848246

[35] Eisfeld, A. J. et al. Pathogenicity and transmissibility of bovine H5N1 influenza virus. *Nature*10.1038/s41586-024-07766-6 (2024). PMC11390473 38977017

[36] Santos, J. J. S. et al. Bovine H5N1 binds poorly to human-type sialic acid receptors. *Nature* **640**, E18–E20 10.1038/s41586-025-08821-6 (2025). 40240859

[37] Herfst, S. et al. Airborne Transmission of Influenza A/H5N1 Virus Between Ferrets. *Science* **336**, 1534–1541 10.1126/science.1213362 (2012). PMC4810786 22723413

[38] Krog, J. S. et al. Influenza A(H10N7) virus in dead harbor seals, Denmark. *Emerg. Infect. Dis.* **21**, 684–687 10.3201/eid2104.141484 (2015). PMC4378493 25811098

[39] Zohari, S., Neimanis, A., Härkönen, T., Moraeus, C. & Valarcher, J. F. Avian influenza A(H10N7) virus involvement in mass mortality of harbour seals (Phoca vitulina) in Sweden, March through October 2014. *Euro Surveill.* **19** 10.2807/1560-7917.es2014.19.46.20967 (2014). 25425511

[40] Herfst, S. et al. Hemagglutinin traits determine transmission of avian A/H10N7 influenza virus between mammals. *Cell Host Microbe* **28**, 602–613.e607 10.1016/j.chom.2020.08.011 (2020). PMC7556738 33031770

[41] Lin, T.-H. et al. A single mutation in bovine influenza H5N1 hemagglutinin switches specificity to human receptors. *Science* **386**, 1128–1134 10.1126/science.adt0180 (2024). PMC12633761 39636969

[42] Agüero, M. et al. Highly pathogenic avian influenza A(H5N1) virus infection in farmed minks, Spain, October 2022. *Eurosurveillance* **28**, 2300001 10.2807/1560-7917.ES.2023.28.3.2300001 (2023). PMC9853945 36695488

[43] Lindh, E. et al. Highly pathogenic avian influenza A(H5N1) virus infection on multiple fur farms in the South and Central Ostrobothnia regions of Finland, July 2023. *Eurosurveillance* **28**, 2300400 10.2807/1560-7917.ES.2023.28.31.2300400 (2023). PMC10401912 37535475

[44] Richard, M. et al. Influenza A viruses are transmitted via the air from the nasal respiratory epithelium of ferrets. *Nat. Commun.* **11**, 766 10.1038/s41467-020-14626-0 (2020). PMC7005743 32034144

[45] Ríos Carrasco, M. et al. The Q226L mutation can convert a highly pathogenic H5 2.3.4.4e virus to bind human-type receptors. *Proc. Natl. Acad. Sci.* **122**, e2419800122 10.1073/pnas.2419800122 (2025). PMC12036971 40232794

[46] Zhang, G. et al. Genomic signatures and host adaptation of H5N1 clade 2.3.4.4b: A call for global surveillance and multi-target antiviral strategies. *Curr. Res. Microb. Sci.* **8**, 100377 10.1016/j.crmicr.2025.100377 (2025). PMC11964551 40177627

[47] Linster, M. et al. Identification, characterization, and natural selection of mutations driving airborne transmission of A/H5N1 Virus. *Cell* **157**, 329–339 10.1016/j.cell.2014.02.040 (2014). PMC4003409 24725402

[48] Xu, D., Tsai, C. J. & Nussinov, R. Hydrogen bonds and salt bridges across protein-protein interfaces. *Protein Eng.* **10**, 999–1012 10.1093/protein/10.9.999 (1997). 9464564

[49] Pace, C. N., Shirley, B. A., McNutt, M. & Gajiwala, K. Forces contributing to the conformational stability of proteins. * FASEB J.* **10**, 75–83 10.1096/fasebj.10.1.8566551 (1996). 8566551

[50] Kumar, S. & Nussinov, R. Close-Range Electrostatic Interactions in Proteins. *ChemBioChem* **3**, 604–617 10.1002/1439-7633(20020703)3:7 (2002). 12324994

[51] Pace, C. N., Scholtz, J. M. & Grimsley, G. R. Forces stabilizing proteins. *FEBS Lett.* **588**, 2177–2184 10.1016/j.febslet.2014.05.006 (2014). PMC4116631 24846139

[52] Chen, J. et al. Structure of the hemagglutinin precursor cleavage site, a determinant of influenza pathogenicity and the origin of the labile conformation. *Cell* **95**, 409–417 10.1016/S0092-8674(00)81771-7 (1998). 9814710

[53] Daniels, R. S. et al. Fusion mutants of the influenza virus hemagglutinin glycoprotein. *Cell* **40**, 431–439 10.1016/0092-8674(85)90157-6 (1985). 3967299

[54] Weis, W. I. et al. The structure of a membrane fusion mutant of the influenza virus haemagglutinin. * EMBO J.* **9**, 17–24 10.1002/j.1460-2075.1990.tb08075.x (1990). PMC551624 2295311

[55] Dadonaite, B. et al. A pseudovirus system enables deep mutational scanning of the full SARS-CoV-2 spike. *Cell* **186**, 1263–1278.e1220 10.1016/j.cell.2023.02.001 (2023). PMC9922669 36868218

[56] Eisen, M. B., Sabesan, S., Skehel, J. J. & Wiley, D. C. Binding of the Influenza A virus to cell-surface receptors: structures of five hemagglutinin–sialyloligosaccharide complexes determined by X-Ray crystallography. *Virology* **232**, 19–31 10.1006/viro.1997.8526 (1997). 9185585

[57] Jurrus, E. et al. Improvements to the APBS biomolecular solvation software suite. *Protein Sci.* **27**, 112–128 10.1002/pro.3280 (2018). PMC5734301 28836357

[58] Dolinsky, T. J., Nielsen, J. E., McCammon, J. A. & Baker, N. A. PDB2PQR: an automated pipeline for the setup of Poisson–Boltzmann electrostatics calculations. *Nucleic Acids Res.* **32**, W665–W667 10.1093/nar/gkh381 (2004). PMC441519 15215472

[59] Hanson, A. et al. Identification of Stabilizing Mutations in an H5 Hemagglutinin Influenza Virus Protein. *J. Virol.* **90**, 2981–2992 10.1128/jvi.02790-15 (2016). PMC4810667 26719265

[60] Singanayagam, A., Zambon, M. & Barclay, W. S. Influenza Virus with Increased pH of Hemagglutinin Activation Has Improved Replication in Cell Culture but at the Cost of Infectivity in Human Airway Epithelium. *Journal of Virology* **93** 10.1128/jvi.00058-00019 (2019). PMC6694820 31189708

[61] Valley, C. C. et al. The Methionine-aromatic Motif Plays a Unique Role in Stabilizing Protein Structure *. *J. Biol. Chem.* **287**, 34979–34991 10.1074/jbc.M112.374504 (2012). PMC3471747 22859300

[62] Mair, C. M. et al. A histidine residue of the influenza virus hemagglutinin controls the pH dependence of the conformational change mediating membrane fusion. *J. Virol.* **88**, 13189–13200 10.1128/jvi.01704-14 (2014). PMC4249083 25187542

[63] Thoennes, S. et al. Analysis of residues near the fusion peptide in the influenza hemagglutinin structure for roles in triggering membrane fusion. *Virology* **370**, 403–414 10.1016/j.virol.2007.08.035 (2008). PMC2212604 17936324

[64] Mueller, D. S. et al. Histidine protonation and the activation of viral fusion proteins. *Biochemical Soc. Trans.* **36**, 43–45 10.1042/bst0360043 (2008). 18208382

[65] Stevens, J. et al. Structure and Receptor Specificity of the Hemagglutinin from an H5N1 Influenza Virus. *Science* **312**, 404–410 10.1126/science.1124513 (2006). 16543414

[66] Kampmann, T., Mueller, D. S., Mark, A. E., Young, P. R. & Kobe, B. The role of histidine residues in low-pH-mediated viral membrane fusion. *Structure* **14**, 1481–1487 10.1016/j.str.2006.07.011 (2006). 17027497

[67] Reed, M. L. et al. Amino acid residues in the fusion peptide pocket regulate the pH of activation of the H5N1 influenza virus hemagglutinin protein. *J. Virol.* **83**, 3568–3580 (2009). 10.1128/JVI.02238-08 PMC2663236 19193808

[68] Zaraket, H., Bridges, O. A. & Russell, C. J. The pH of activation of the hemagglutinin protein regulates H5N1 influenza virus replication and pathogenesis in mice. *J. Virol.* **87**, 4826–4834 10.1128/JVI.03110-12 (2013). PMC3624295 23449784

[69] Blumenkrantz, D., Roberts, K. L., Shelton, H., Lycett, S. & Barclay, W. S. The short stalk length of highly pathogenic avian influenza H5N1 virus neuraminidase limits transmission of pandemic H1N1 virus in ferrets. *J. Virol.* **87**, 10539–10551 10.1128/jvi.00967-13 (2013). PMC3807409 23864615

[70] Yu, T. C. et al. Pleiotropic mutational effects on function and stability constrain the antigenic evolution of influenza haemagglutinin. *Nat. Ecol. Evol.* **10**, 452–466 10.1038/s41559-025-02895-1 (2026). PMC12758803 41326606

[71] Ahn, J. J., Yu, T. C., Dadonaite, B., Radford, C. E. & Bloom, J. D. Influenza hemagglutinin subtypes have different sequence constraints despite sharing extremely similar structures. *Virus Evol.* **12** 10.1093/ve/veag018 (2026). PMC13064934 41971714

[72] Dosey, A. et al. Stabilization of H5 highly pathogenic avian influenza hemagglutinin improves vaccine-elicited neutralizing antibody responses. *bioRxiv*, 2025.2007.2030.667762 (2025) 10.1101/2025.07.30.667762.

[73] Yu, T. C. et al. Pleiotropic mutational effects on function and stability constrain the antigenic evolution of influenza haemagglutinin. *Nat. Ecol. Evol.* 10.1038/s41559-025-02895-1 (2025). PMC12758803 41326606

[74] Manivannan, S. N., Diaz Arenas, C., Grubaugh, N. D. & Ogbunugafor, C. B. The importance of epistasis in the evolution of viral pathogens. *Virus Evol.* **11** 10.1093/ve/veaf091 (2025). PMC12694738 41383811

[75] Wang, L. et al. Impact of naturally occurring hemagglutinin substitutions on antigenicity and fitness of influenza A(H5N1) virus. *npj Viruses* **3**, 72 10.1038/s44298-025-00154-5 (2025). PMC12489106 41034380

[76] Abu-Shmais, A. A. et al. Cross-neutralizing and potent human monoclonal antibodies against historical and emerging H5Nx influenza viruses. *Nat. Microbiol.* **10**, 2903–2918 10.1038/s41564-025-02137-x (2025). PMC12578633 41087744

[77] Nguyen, Y. T. K. et al. Structural characterization of influenza group 1 chimeric hemagglutinins as broad vaccine immunogens. *Proc. Natl. Acad. Sci.* **122**, e2416628122 10.1073/pnas.2416628122 (2025). PMC11848309 39937865

[78] Zhu, X. et al. Influenza chimeric hemagglutinin structures in complex with broadly protective antibodies to the stem and trimer interface. *Proc. Natl. Acad. Sci.* **119**, e2200821119 10.1073/pnas.2200821119 (2022). PMC9173763 35594401

[79] Milder, F. J. et al. Universal stabilization of the influenza hemagglutinin by structure-based redesign of the pH switch regions. *Proc. Natl. Acad. Sci.* **119**, e2115379119 10.1073/pnas.2115379119 (2022). PMC8833195 35131851

[80] Lee, C.-Y. et al. Epistasis reduces fitness costs of influenza A virus escape from stem-binding antibodies. *Proc. Natl. Acad. Sci.* **120**, e2208718120 10.1073/pnas.2208718120 (2023). PMC10151473 37068231

[81] Rudneva, I. et al. Escape mutants of pandemic influenza A/H1N1 2009 virus: Variations in antigenic specificity and receptor affinity of the hemagglutinin. *Virus Res.* **166**, 61–67 10.1016/j.virusres.2012.03.003 (2012). 22459010

[82] Lee, N. et al. The use of plant lectins to regulate H1N1 influenza A virus receptor binding activity. *PLOS ONE* **13**, e0195525 10.1371/journal.pone.0195525 (2018). PMC5891020 29630683

[83] Lei, R. et al. Epistasis mediates the evolution of the receptor binding mode in recent human H3N2 hemagglutinin. *Nat. Commun.* **15**, 5175 10.1038/s41467-024-49487-4 (2024). PMC11189414 38890325

[84] McAuley, J. L., Gilbertson, B. P., Trifkovic, S., Brown, L. E. & McKimm-Breschkin, J. L. Influenza Virus Neuraminidase Structure and Functions. *Front. Microbiol.* **10** 10.3389/fmicb.2019.00039 (2019). PMC6362415 30761095

[85] de Vries, E., Du, W., Guo, H. & de Haan, C. A. M. Influenza A Virus Hemagglutinin–Neuraminidase–Receptor Balance: Preserving Virus Motility. *Trends Microbiol.* **28**, 57–67 10.1016/j.tim.2019.08.010 (2020). PMC7172302 31629602

[86] Yang, X. et al. A Beneficiary Role for Neuraminidase in Influenza Virus Penetration through the Respiratory Mucus. *PLOS ONE* **9**, e110026 10.1371/journal.pone.0110026 (2014). PMC4198190 25333824

[87] Cohen, M. et al. Influenza A penetrates host mucus by cleaving sialic acids with neuraminidase. *Virol. J.* **10**, 321 10.1186/1743-422X-10-321 (2013). PMC3842836 24261589

[88] Iseli, A. N. et al. The neuraminidase activity of influenza A virus determines the strain-specific sensitivity to neutralization by respiratory mucus. *J. Virol.* **97**, e01271–01223 10.1128/jvi.01271-23 (2023). PMC10617592 37819131

[89] Vahey, M. D. & Fletcher, D. A. Influenza A virus surface proteins are organized to help penetrate host mucus. *eLife* **8**, e43764 10.7554/eLife.43764 (2019). PMC6516830 31084711

[90] Müller, M., Lauster, D., Wildenauer, H. H. K., Herrmann, A. & Block, S. Mobility-based quantification of multivalent virus-receptor interactions: new insights into influenza A virus binding mode. *Nano Lett.* **19**, 1875–1882 10.1021/acs.nanolett.8b04969 (2019). 30719917

[91] Mitnaul, L. J. et al. Balanced hemagglutinin and neuraminidase activities are critical for efficient replication of influenza A virus. *J. Virol.* **74**, 6015–6020 10.1128/jvi.74.13.6015-6020.2000 (2000). PMC112098 10846083

[92] Xu, R. et al. Functional balance of the hemagglutinin and neuraminidase activities accompanies the emergence of the 2009 H1N1 Influenza Pandemic. *J. Virol.* **86**, 9221–9232 10.1128/jvi.00697-12 (2012). PMC3416152 22718832

[93] Liu, T., Wang, Y., Tan, T. J. C., Wu, N. C. & Brooke, C. B. The evolutionary potential of influenza A virus hemagglutinin is highly constrained by epistatic interactions with neuraminidase. *Cell Host Microbe* **30**, 1363–1369.e1364 10.1016/j.chom.2022.09.003 (2022). PMC9588755 36150395

[94] Ilyushina, N. A., Bovin, N. V. & Webster, R. G. Decreased neuraminidase activity is important for the Adaptation of H5N1 influenza virus to human airway epithelium. *J. Virol.* **86**, 4724–4733 10.1128/jvi.06774-11 (2012). PMC3347354 22379077

[95] Hermann, E. & Krammer, F. Clade 2.3.4.4b H5N1 neuraminidase has a long stalk, which is in contrast to most highly pathogenic H5N1 viruses circulating between 2002 and 2020. *mBio* **16**, e03989–03924 10.1128/mbio.03989-24 (2025). PMC11980542 40008897

[96] Giannecchini, S. et al. Comparison of in vitro replication features of H7N3 influenza viruses from wild ducks and turkeys: potential implications for interspecies transmission. *J. Gen. Virol.* **87**, 171–175 10.1099/vir.0.81187-0 (2006). 16361429

