# Supplementary material for: In silico analysis of pH stabilising mutations of hemagglutinin of influenza A virus H5N1 clade 2.3.4.4b
Source: Npj Viruses. 2026 Sep 25;4:44. doi: 10.1038/s44298-026-00236-y (PMC13614952; doi:10.1038/s44298-026-00236-y)
Supplement: Supplementary file 1 [file 44298_2026_236_MOESM1_ESM.pdf]

Supplementary Information

In silico Analysis of pH Stabilising Mutations of Hemagglutinin of Influenza A Virus H5N1 clade 2.3.4.4b

Daniel Christian Lauster[1#], Christian Sieben[2#], Matthias Ballauff[3], Andreas Herrmann[3]

[1]Freie Universität Berlin, Institut für Pharmazie, Biopharmazeutika, 14195 Berlin, Germany

[2]Nanoinfektionsbiologie (NIBI), HZI - Helmholtz-Zentrum für Infektionsforschung GmbH, 38124 Braunschweig, Germany

[3]Freie Universität Berlin, Institut für Chemie und Biochemie, SupraFAB, 14195 Berlin, Germany

# shared first authorship and corresponding authors:

Email: daniel.lauster@fu-berlin.de; Christian.Sieben@helmholtz-hzi.de

Scheme S1. Sequences H5 American-Wigeon South Carolina 2021 (analysed by Dadonaite et al. (2024)) with H5 A/Texas/37/2024 (pdb 9DIP). The HA2 sequences of the transmembrane domain and the intraviral tail were removed for methodological reasons. (Mutations are coloured, see Methods)

HA1

American-Wigeon South Carolina 2021
LVKSDQICIGYHANNSTEQVDTIMEKNVTVTHAQDILEKTHNGKLCDLNG
VKPLILKDCSVAGWLLGNPMCDEFIRVPEWSYIVERANPANDLCYPGSLNDYEELKHMLSRI
NHFEKILIIPKSSWPNHETSLGVSAACPYQGAPSFFRNVVWLIKKNDAYPTIKISYNNTNRE
DLLILWGIHHSNNAEEQTNLYKNPTTYISVGTSTLNQRLAPKIATRSQVNGQRGRMDFFWTI
LKPDDAIHFESNGNFIAPEYAYKIVKKGDSTIMKSGVEYGHCNTKCQTPVGAINSSMPFHNI
HPLTIGECPKYVKSNKLVLATGLRNSPLREKRRKR

A/Texas/37/2024
ADPGDQICIGYHANNSTEQVDTIMEKNVTVTHAQDILEKTHNGKLCDLNG
VKPLILKDCSVAGWLLGNPMCDEFIRVPEWSYIVERANPANDLCYPGSLNDYEELKHMLSRI
NHFEKIQIIPKSSWPNHETSLGVSAACPYQGAPSFFRNVVWLIKKNDAYPTIKISYNNTNRE
DLLILWGIHHSNNAEEQTNLYKNPITYISVGTSTLNQRLAPKIATRSQVNGQRGRMDFFWTI
LKPNDAIHFESNGNFIAPEYAYKIVKKGDSTIMKSGVEYGHCNTKCQTPVGAINSSMPFHNI
HPLTIGECPKYVKSNKLVLATGLRNSP

HA2

American-Wigeon South Carolina 2021
GLFGAIAGFIEGGWQGMVDGWYGYHHSNEQGSGYAADKESTQKAIDGVTNKVNSIIDKMNTQ
FEAVGREFNNLERRIENLNKKMEDGFLDVWTYNAELLVLMENERTLDFHDSNVKNLYDKVRL
QLRDNAKELGNGCFEFYHKCDNECMESVRNGTYDYPQYSEEARLKREEISGVKLESVGTYQI
LSIYSTAASSLALAIMMAGLSLWMCSNGSLQCRICI*

A/Texas/37/2024
GLFGAIAGFIEGGWQGMVDGWYGYHHSNEQGSGYAADKESTQKAIDGVTNKVNSIIDKMNTQ
FEAVGREFNNLERRIENLNKKMEDGFLDVWTYNAELLVLMENERTLDFHDSNVKNLYDKVRL
QLRDNAKELGNGCFEFYHKCDNECMESVRNGTYDYPQYSEEARLKREEISSG

Table S1 Overview of destabilising mutations in the ectodomain and their location of influence, i.e. whether within the subunit, between the two subunits of a monomer and/or between monomers, as well as a possible influence on the surface potential of the trimer (indicated by √).

Sub-unit	Mutation	Influence of Mutation			
		**Within subunit**	**Intramonomer HA1-HA2 interface**	**Intermonomer interface**	**Surface potential ectodomain**
HA1	His184...			HA1-HA1	
	Arg210...			HA1-HA1	
	Arg220...			HA1-HA1	
	Asp241...			HA1-HA1	
	Lys307...		√	HA1-HA2	
	Thr318			HA1-HA2	
	Gly319			HA1-HA2	
HA2	Tyr22$_{(2)}$	√		HA1-HA2	
	Lys51$_{(2)}$	√			
	Lys58$_{(2)}$...			-	
	Asn60$_{(2)}$...		√	HA2-HA2	
	Glu74$_{(2)}$...			HA2-HA2	
	Arg76$_{(2)}$...			HA2-HA2	
	Asn81($_{2)}$			HA2-HA2	
	Lys82$_{(2)}$			HA2-HA2	
	Lys83$_{(2)}$			HA2-HA2	
	Asp90$_{(2)}$...			HA2-HA2	
	Glu97$_{(2)}$...			HA2-HA2	
	Gly103$_{(2)}$	√			
	Thr107$_{(2)}$	√			
	Arg106$_{(2)}$			HA2-HA2	
	Asn114$_{(2)}$	√		HA2-HA2	
	Arg123$_{(2)}$...			HA2-HA2	
	Glu132$_{(2)}$...			HA2-HA2	
	His111$_{(2)}$...	√		HA1-HA2	√

Figure S1

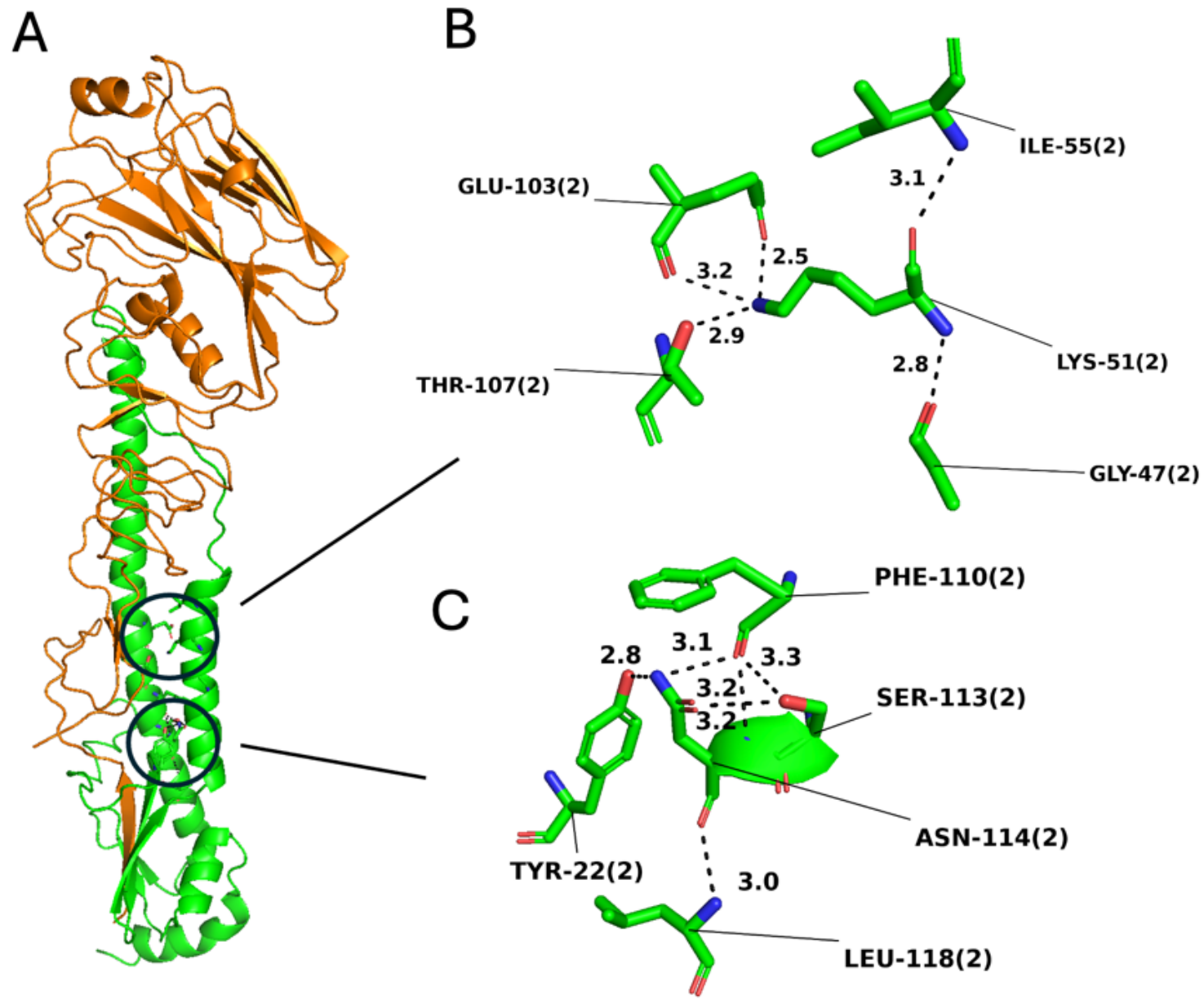

Figure S1. The residues Lys51$_{(2)}$ and Asn114$_{(2)}$ each form several polar bonds between the two parallel HA2 helices of a monomer. Mutations of these residues have a detrimental effect on the stability of HA.

Lys51$_{(2)}$: This residue is highly preserved among subtypes[1-3]. Lys51$_{(2)}$ appears to play an important role in the stability of the HA2 subunit, as any mutation reduces the stability of the conformation[4]. Located in the short helix of the HA2 stem region, Lys51$_{(2)}$ has polar contacts to residues within the helix (Gly47$_{(2)}$ and Ile55$_{(2)}$) as well as with residues Glu103$_{(2)}$ and Thr107$_{(2)}$ of the long helix of this subunit (Figure S1). These residues emphasise the importance of the polar contacts to Lys51$_{(2)}$. For Thr107$_{(2)}$, as well as for Gly47$_{(2)}$ and Ile55$_{(2)}$ only destabilizing mutations were found. For Glu103$_{(2)}$, only mutations to Asp or Ser led to slightly increased stability in contrast to other mutations (Dadonaite et al. (2024)[4]).

Arg106$_{(2)}$: The residue Arg106$_{(2)}$ in the coiled-coil domain of the HA2 stem region (not shown) is characteristic of the H5 subtype. In case of H5N1 clade2.3.4.4b, there are polar interactions of Arg106$_{(2)}$ with Met102$_{(2)}$ and Phe110$_{(2)}$ within the same helix, as well as with Glu105$_{(2)}$ within the helix of the neighbouring HA2 subunit. Although Dadonaite et al. (2024) found mutations of Arg106$_{(2)}$ generally destabilizing, mutations to Glu or Gln increased stability - but without improved pseudovirus entry in 293T cells, highlighting the complexity of predicting mutation effects.

Asn114$_{(2)}$: Next to the network around Lys51$_{(2)}$, a further polar contact is established between the two neighbouring HA2 helices via Asn114$_{(2)}$ and Tyr22$_{(2)}$ (see Figure S1C). This contact also appears to be important for stability, as any mutation of Tyr22$_{(2)}$ leads to destabilization[4]. Mutations of Asn114$_{(2)}$ are similarly sensitive to stability.

Figure S2

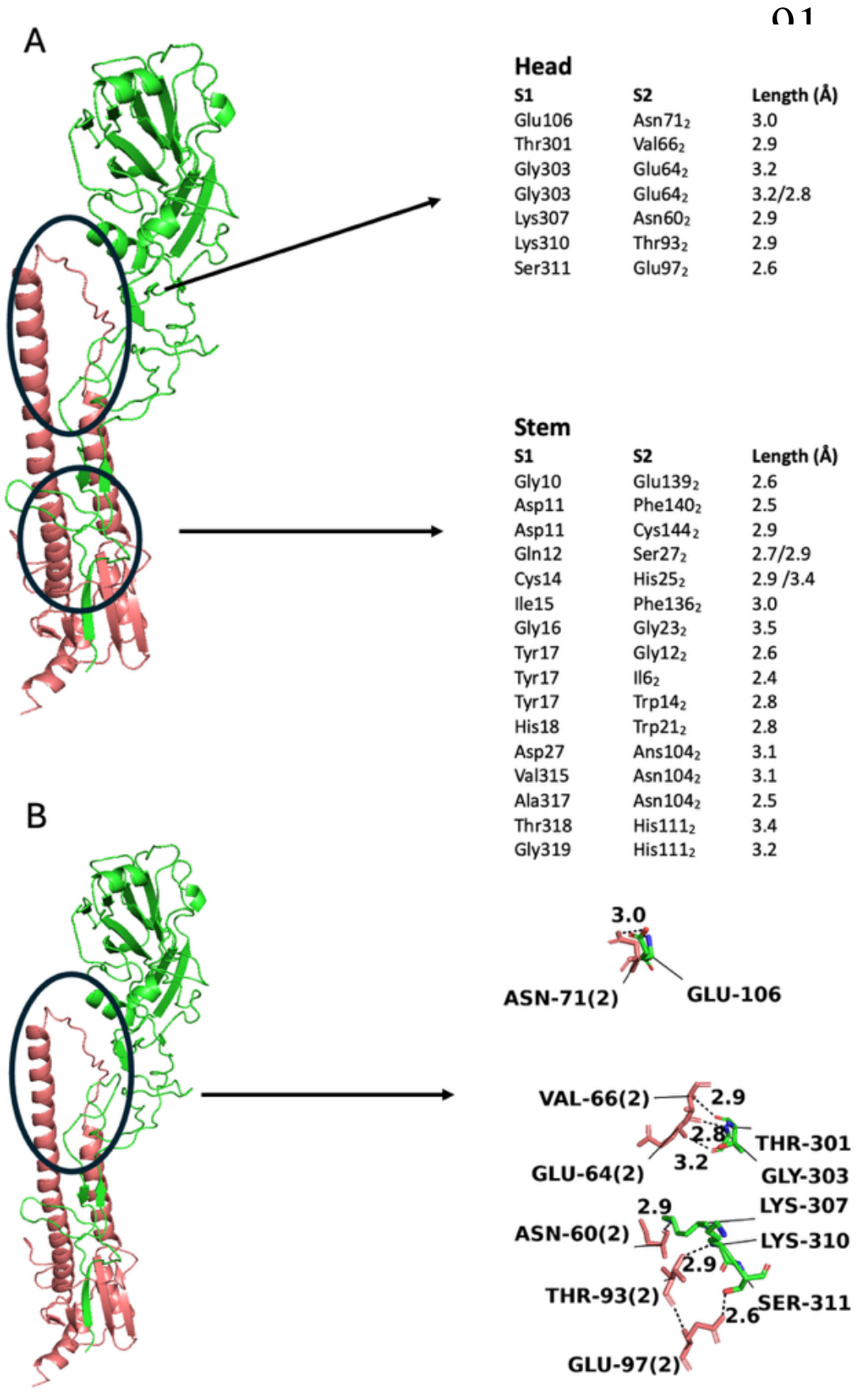

Head

S1	S2	Length (Å)
Glu106	$Asn71_2$	3.0
Thr301	$Val66_2$	2.9
Gly303	$Glu64_2$	3.2
Gly303	$Glu64_2$	3.2/2.8
Lys307	$Asn60_2$	2.9
Lys310	$Thr93_2$	2.9
Ser311	$Glu97_2$	2.6

Stem

S1	S2	Length (Å)
Gly10	$Glu139_2$	2.6
Asp11	$Phe140_2$	2.5
Asp11	$Cys144_2$	2.9
Gln12	$Ser27_2$	2.7/2.9
Cys14	$His25_2$	2.9 /3.4
Ile15	$Phe136_2$	3.0
Gly16	$Gly23_2$	3.5
Tyr17	$Gly12_2$	2.6
Tyr17	$Il6_2$	2.4
Tyr17	$Trp14_2$	2.8
His18	$Trp21_2$	2.8
Asp27	$Ans104_2$	3.1
Val315	$Asn104_2$	3.1
Ala317	$Asn104_2$	2.5
Thr318	$His111_2$	3.4
Gly319	$His111_2$	3.2

Figure S2. Overview on polar interactions between the HA1 and HA2 subunits analysed using PyMOL. (A) HA1-HA2 interactions in the lower part of the HA1 head domain und in the stem region. (B) Zooming into the interaction network in the lower part of the HA1 head domain.

The figure illustrates the numerous polar interactions of the amino acids that stabilise the inner surface of an HA1-HA2 monomer. There are many interactions in both the head and stem regions. Figure S2B shows the individual interactions within the head region. The numerous HA1-HA2 interactions in the stem region demonstrate the stability of the stem region and its high sensitivity to mutations. Stability only increases significantly in a few cases; for example, in the head region of the HA1-HA2 interface, the Glu106Asp mutation occurs. However, this extensive network of stabilising interactions could eventually counteract individual mutations that would otherwise have a detrimental effect on stability.

Figure S3

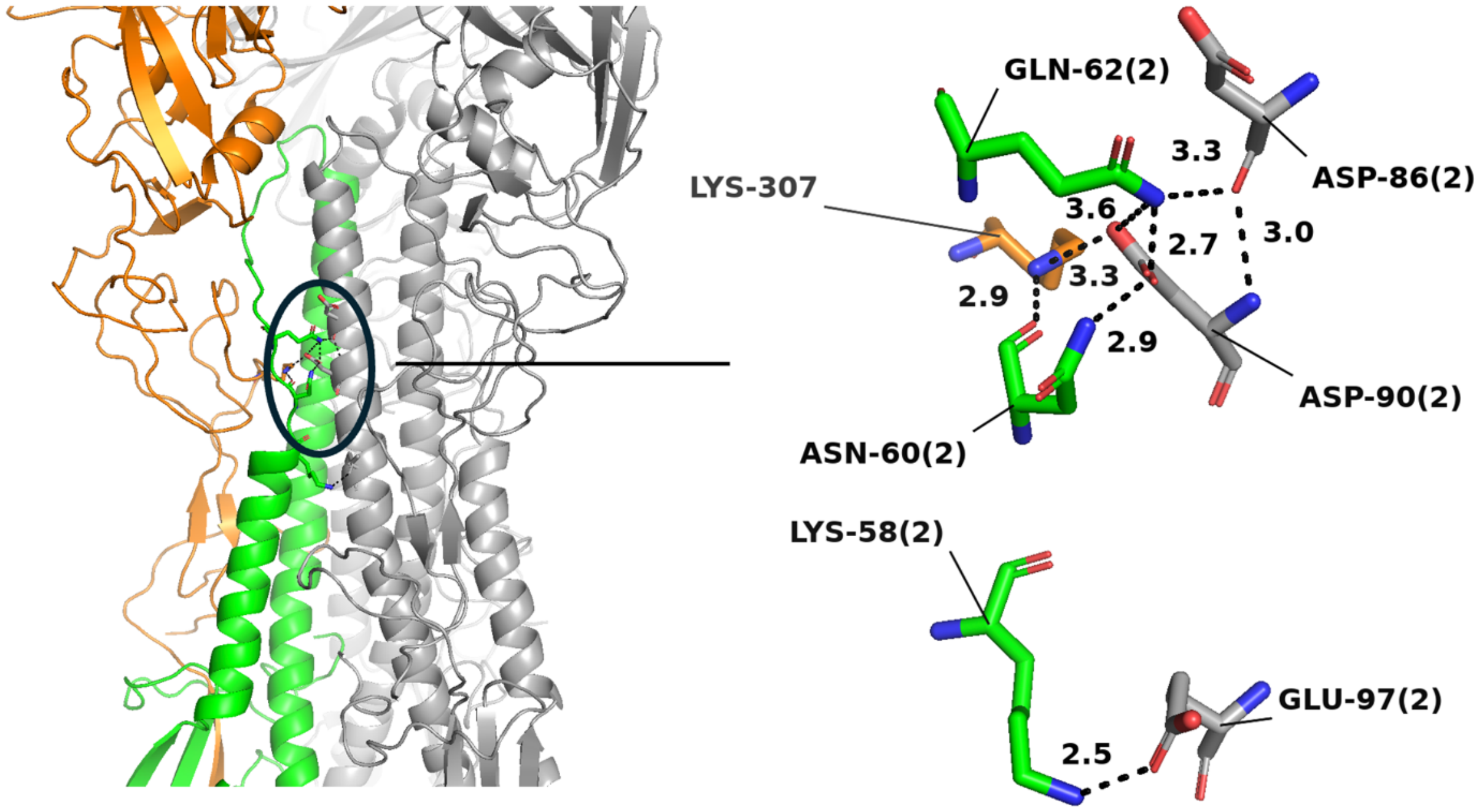

Figure S3. Network of polar interactions of Lys307 and Lys58$_{(2)}$ with residues of the HA2 subunit of the same monomer (green) and the HA2 subunit of the neighbouring HA2 subunit (grey).

Lys307: The results of Dadonaite et al. (2024)[4] suggest that the amino acid Lys307 of the HA1 subunit plays a significant role in the HA stability, as any characterised mutation has negative consequences. The analysis shows that Lys307 forms contacts both with both Asn60$_{(2)}$ within the monomer and with Asp90$_{(2)}$ of the HA2 subunit of the neighbouring monomer (Figure S3).

Asn60$_{(2)}$: Asn60$_{(2)}$ is integrated into a complex network of amino acids (see Figure S3). It interacts with Lys307 of HA1 of the same monomer, as well as with Asp90$_{(2)}$ of the long helix of an adjacent HA2 monomer. Additionally, Lys307 and Gln62$_{(2)}$ of a monomer are in contact with Asp86$_{(2)}$ and Asp90$_{(2)}$ of an adjacent monomer. The mutations Asn60$_{(2)}$Val and Asn60$_{(2)}$Ile only lead to a slight increased stability (Dadonaite et al., (2024)[4]). For Asn60$_{(2)}$Ile, the intramonomeric contact with Lys307 is maintained; however, both mutations result in a loss of contact with the neighbouring monomer. The entry of both mutations into 293T cells is only slightly reduced. All other mutations of Asn60$_{(2)}$ lead to destabilization.

Asp90$_{(2)}$, Asp86$_{(2)}$ and Gln62$_{(2)}$: These three residues are located in the central part of the stem region (not shown). In addition to interacting with the HA1 subunit (see above), Asp90$_{(2)}$ interacts with both Asp86$_{(2)}$ and Gln62$_{(2)}$, the former being located within the same HA2 subunit and the latter within the neighbouring HA2 subunit in the vicinity of Asn60$_{(2)}$. The importance of Asp90$_{(2)}$ for HA stability is demonstrated by its susceptibility to mutation, except for the Asp90$_{(2)}$Glu mutation[4], which retains the site's negative charge.

Asn114$_{(2)}$: Residue Asn114$_{(2)}$ is located in the helical region of HA2 proximal to the viral envelope (not shown) and interacts with Tyr22$_{(2)}$ of the neighbouring HA2 helix (not shown). Deep mutational analysis revealed that any mutation to this residue is disadvantageous for stability[4].

Figure S4

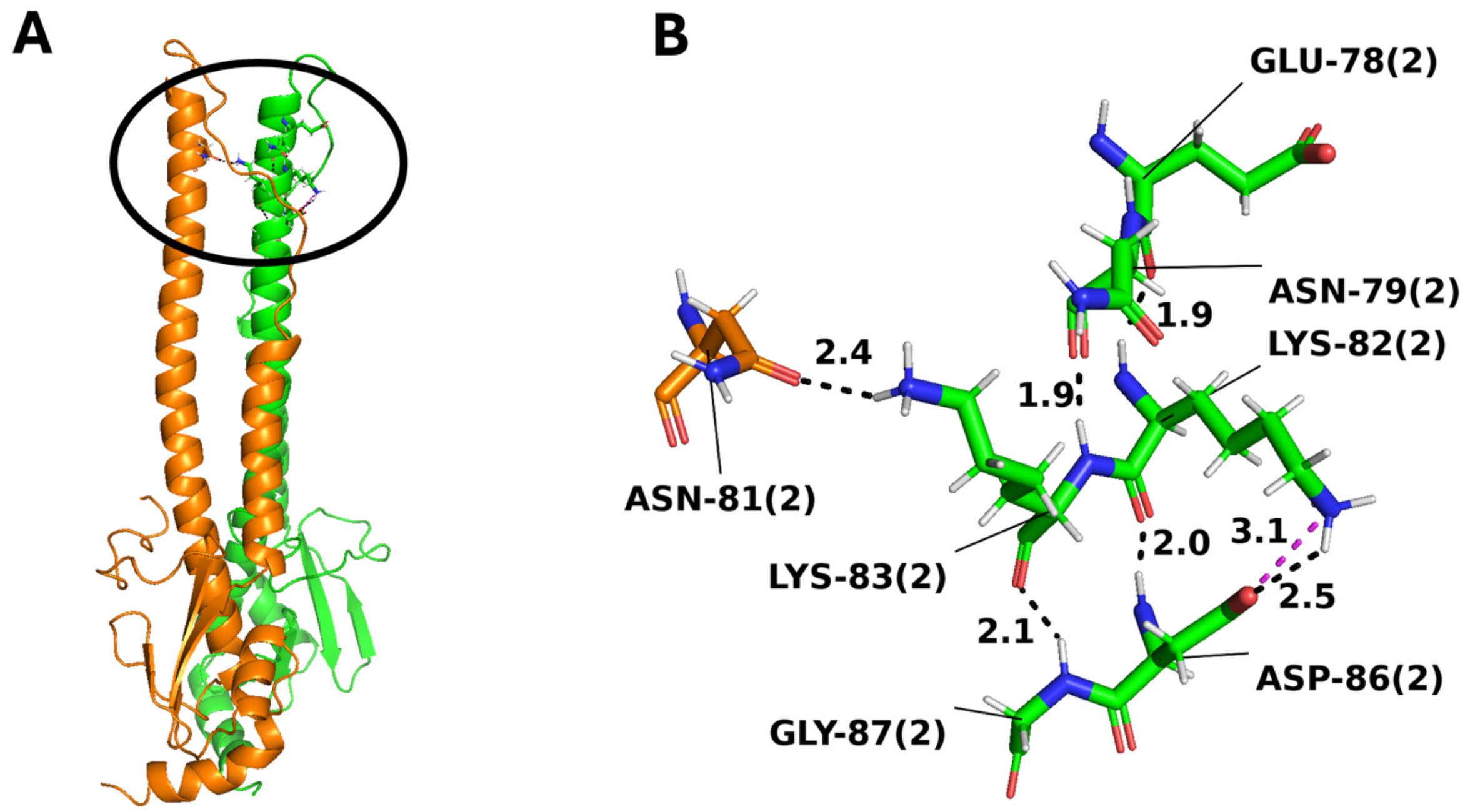

Figure S4. The intra- and intersubunit polar network of residues of Lys82$_{(2)}$ and Lys83$_{(2)}$. Lys83$_{(2)}$ interacts with Ans81(2) of the neighbouring HA2 subunit (subunits in green and in orange). (A) Localisation of the network. (B) Interaction between residues of both HA2 subunits. Magenta: salt bridge between Asp86(2) and Lys82(2).

Lys82$_{(2)}$ and Lys83$_{(2)}$: Lys82$_{(2)}$, Lys83$_{(2)}$, and Asn81$_{(2)}$ are located at the top of the long HA2 helix bundle within the region of high positive surface potential (Figure 5). Lys83$_{(2)}$ forms polar interactions with the neighbouring HA2 subunit via Asn81$_{(2)}$ and, via Lys82$_{(2)}$, with an intrasubunit network (Figure S4). Only destabilising mutations were identified for Lys83$_{(2)}$, and only few non-destabilising mutations occur at Lys82$_{(2)}$ [4]. For Asn81$_{(2)}$, only the Glu substitution is stabilising; it maintains intersubunit interaction via Arg68$_{(2)}$ in the loop connecting the helices. The low mutational tolerance of Lys82$_{(2)}$, Lys83$_{(2)}$, and Asn81$_{(2)}$ underscore their importance for the ECD stability.

Figure S5

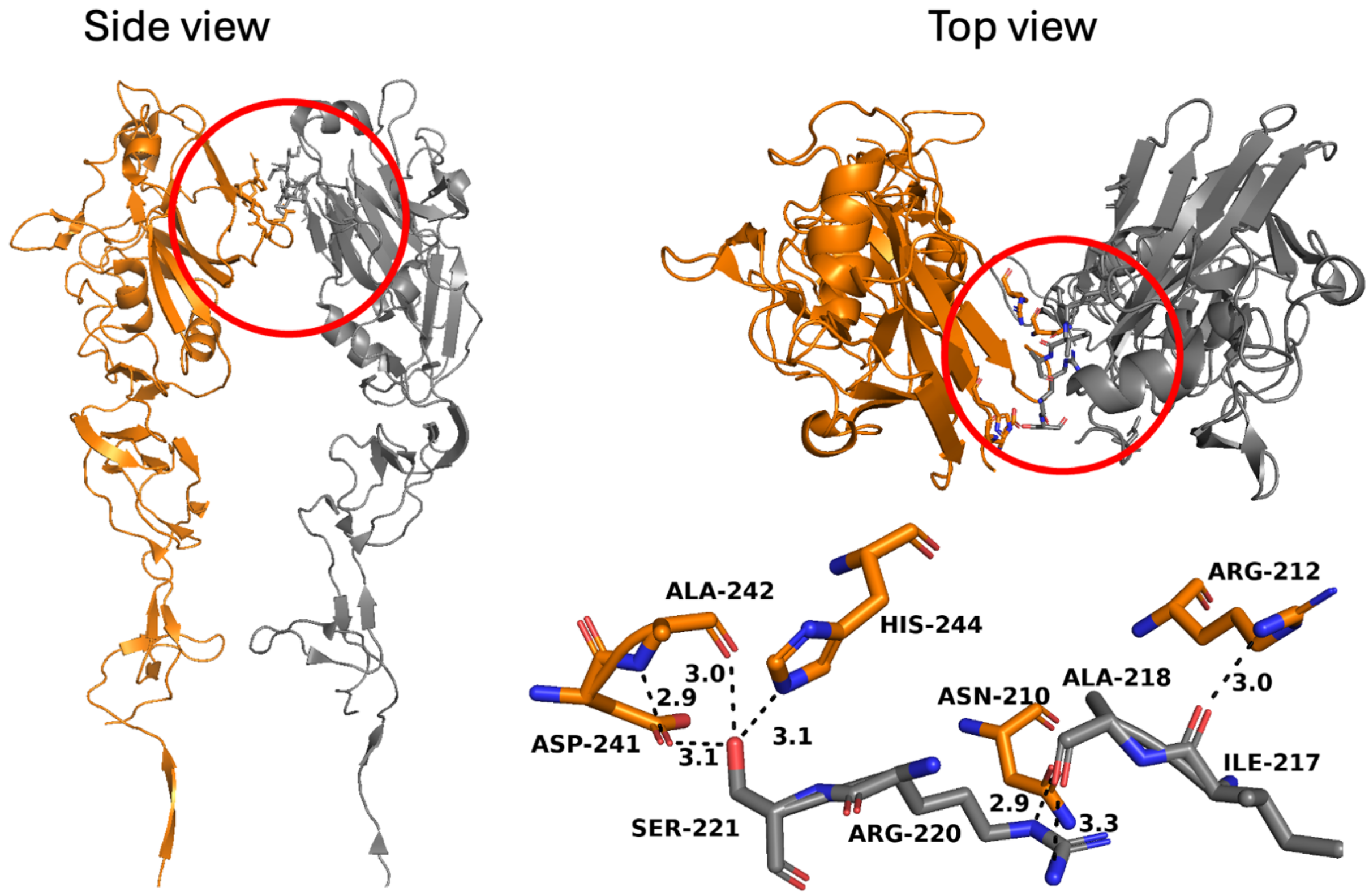

Figure S5. Polar interactions within the HA1 head domains. An overview is shown in 'Side view' and 'Top view'. Lower right: Zooming into the interaction network.

There are several polar contacts between the head domains of HA1 subunits. Ser221 and Arg220 in one HA1 subunit interact with residues Asp241, Ala242, His244 and Asn210 in the neighbouring HA1 subunit, respectively. There is also a polar contact between Arg212 and Ile217. The stability of the ECD is impaired by mutations of these residues involved, particularly to mutations Arg210, Arg220 and Asp241 (Dadonaite et al. (2024)[4]). This is also reflected by a significantly reduced uptake in 293T cells[4]. According to our analysis using PyMOL, salt bridges are not involved.

Figure S6

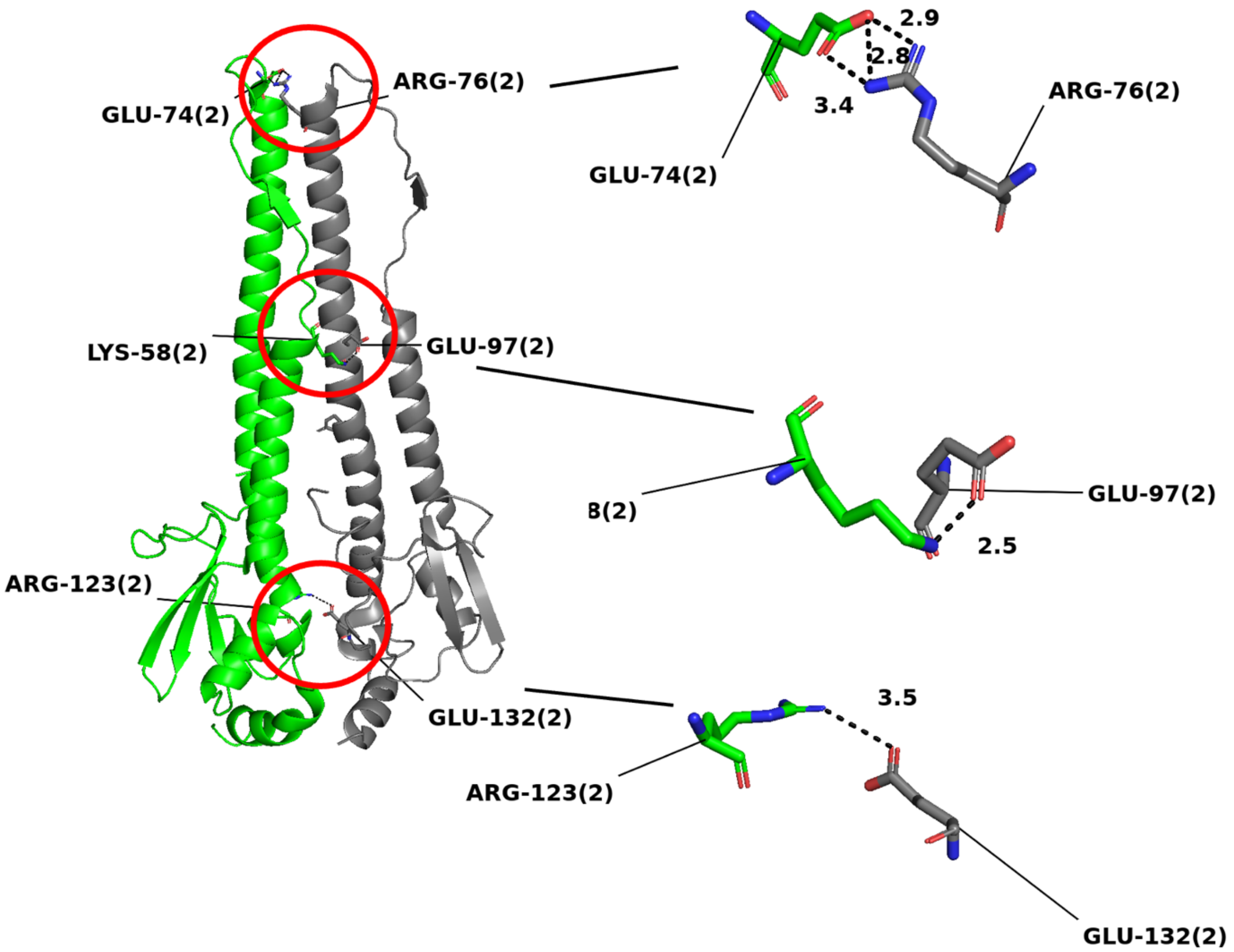

Figure S6. Salt bridges between two HA2 subunits. For details see below.

According to our analysis, there are three salt bridges between the long helices of two HA2 subunits (Figure S6). These are localized in the lower (Arg123$_{(2)}$-Glu132$_{(2)}$), middle (Lys58$_{(2)}$-Glu97$_{(2)}$) and upper (Glu74$_{(2)}$-Arg76$_{(2)}$) regions. In general, mutations that disrupt salt bridges in the upper and lower regions do not positively affect HA stability at an acidic pH[4]. For Arg123$_{(2)}$, only mutations by Lys and His preserve the stability as can be inferred from the data of Dadonaite et al. (2024)[4]. Therefore, we conclude that the presence and location of these two salt bridges in the long helices of HA2 are important for the stability of the stem region. This is also reflected by the fact that all measured mutations negatively affect entry into 293T cells.

Figure S7

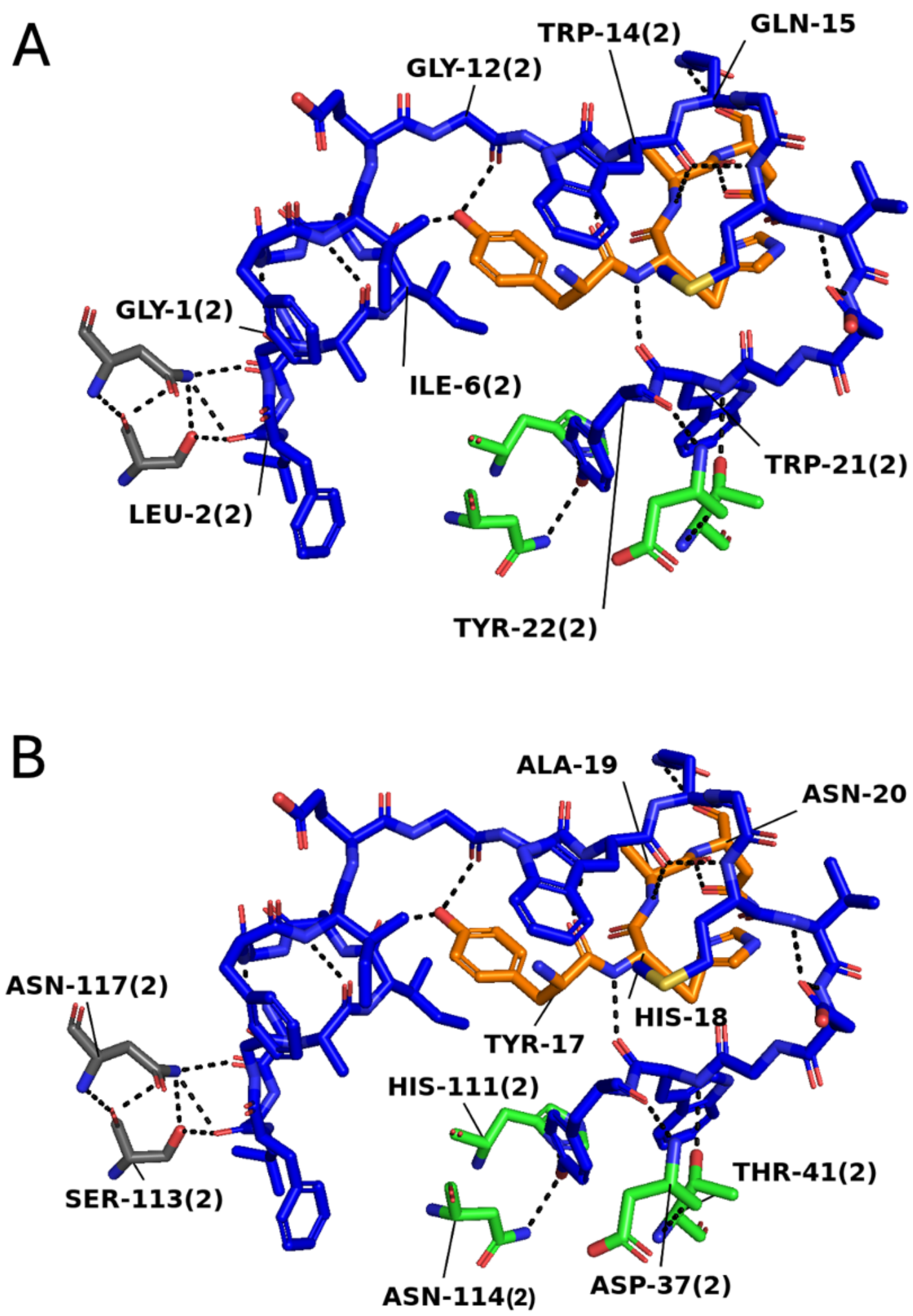

Figure S7. Interaction network of the fusion peptide**.** Polar interactions of the fusion peptide residues with neighbouring residues of the HA2 subunits and the HA1 subunit (fusion peptide – blue; residues of the same HA2 subunit – green; residues of the HA1 subunit of the monomer – orange; HA2 subunit of the neighbouring monomer – grey). (A) Residues of the fusion peptide are labelled. (B) Residues of the residues interacting with the fusion peptide are labelled.

About the first 20 amino acids of the HA2 N terminus form the fusion peptide. In particular, the first 10 residues and some residues in the pocket are highly conserved between the influenza virus subtypes. In general, amino acid mutations or deletions in both the peptide and the pocket have been shown to be associated with an impairment or loss of fusion activity implicating interactions between respective residues [1,2,5-8] .

We identified several polar contacts in the H5 clade 2.3.4.4b HA between residues of the fusion peptide and residues of both the HA1 and HA2 subunits of the same monomer, as well as between the HA2 subunits of neighbouring monomers (Figure S7). The distances of the polar contacts are not greater than 3.4 Å, typically less than 3.0 Å. The analysis revealed no evidence for salt bridges. Mutations of the $Gly12_{(2)}$, $Trp14_{(2)}$, $Trp21_{(2)}$ and $Tyr22_{(2)}$ residues on the fusion peptide are detrimental to stability. This also applies to the fusion peptide interacting residues $Asp37_{(2)}$, $Thr41_{(2)}$, His $111_{(2)}$ and $Asn114_{(2)}$ of the same HA2 subunit and

Asn20 of the HA1 subunit of the same monomer. Mutation of His18 to phenylalanine or glutamine led to stabilization of the HA (see above). This is consistent with studies by Reed et. (2009)[2], which showed that mutation to glutamine lowered the fusion pH by 0.3 units. Mutations of $Ala19_1$ of the latter subunit have neither a negative nor a significant positive effect on stability. Furthermore, several mutations of $Ser113_{(2)}$ and $Asn117_{(2)}$ of the neighbouring HA2 subunit promote the stability of the ECD. It is noteworthy that the majority of mutations in $Gly4_{(2)}$ may have a stabilising effect. The $Ala5_{(2)}$Met mutation has a highly stabilising effect on the ECD; however, all other $Ala5_{(2)}$ mutations are detrimental. However, a comparison of influenza virus subtypes shows that the first 14 amino acids of the fusion peptide, in particular, are highly conserved and that any stabilising mutations may not have manifested themselves. Clearly, the function of the fusion peptide – interaction with the target membrane to be fused – determines its conserved sequence.

Tyr17: This residue is located in the fusion peptide pocket (see Figure S7B). Tyr17 forms polar contacts with the HA2 subunit of the same monomer via $Ile6_{(2)}$, $Gly12_{(2)}$ and $Trp14_{(2)}$. Dadonaite et al. (2024)[4] observed a slight increase in the stability for the Tyr17His mutation. As these polar contacts are preserved by His, it is possible that its more polar character could be responsible for the increased stability. Notably, the stabilizing mutation Tyr17His has also been identified in HA of subtype H3. A reduction in fusion pH of 0.3 was observed[1,6]. In contrast, an increase of 0.4 units was reported for H5 strains[2,9,10], indicating destabilization of the HA prefusion conformation. Obviously, the influence of this mutation on stability is strain-specific. Importantly, the Dadonaite et al. (2024)[4] data show that the stabilizing effect was much more pronounced for the Met and Thr mutations of Tyr17. The polar contacts of Thr with both His18 and $Trp21_{(2)}$ clearly stabilize the loop of the fusion peptide. (see chapter 'Interaction Network of the Fusion Peptide').

The residues $Asp109_{(2)}$ and $Asp112_{(2)}$ (not shown) are conserved among IAV subtypes except for subtype H17[1]. Weis et al. (1990) reported for HA of A/31 (H3N2) that $Asp109_{(2)}$ and $Asp112_{(2)}$ form hydrogen bonds with several residues of the fusion peptide[11]. Mutation $Asp109_{(2)}$Gly caused local destabilization due a loss of hydrogen bonds and thereby the exposure of the fusion peptide at higher pH[11]. In the PyMOL analysis of the 3D structure of A/X31 (pdb 2VIU), we also found hydrogen bonds for both residues, but not for H5 HA of A/Texas/37/2024 (pdb 9DIP). The analysis by Dadonaite et al. (2024)[4] also shows that several amino acids can replace $Asp109_{(2)}$ causing in few cases a slightly increased stability if it all. However, the mutation $Asp109_{(2)}$Gly was missing in the analysis. Although highly conserved, many mutations of $Asp112_{(2)}$ caused a significant stabilization of the HA structure[4].

Reed et al. (2009)[2] reported for HA of A/chicken/Vietnam/C58/04 (H5N1) that the mutation $His111_{(2)}$Ala led to a loss of fusion activity. In our analysis (see *'Histidine Residues as pH Sensors for the HA Conformational Change'*) we found a polar interaction of $His111_{(2)}$ with $Tyr22_{(2)}$, and Gly319 and Thr318 of the neighbouring monomer. Indeed, the deep mutational scanning showed that mutation of these residues are highly sensitive to all amino acids causing a destabilization of the stability[4].

Reed et al. (2010)[10] found for the same strain that a mutation in the immediate vicinity - $Asn114_{(2)}$Lys - resulted in an increase in the pH value triggering the structural change of + 0.5 resulting in a decreased replication and environmental stability. The mutation was genetically

unstable: after several passage the mutation was reversed. The analysis of Dadonaite et al. (2024)[4] confirmed that the stability was decreased by mutation of Asn114$_{(2)}$.

Figure S8

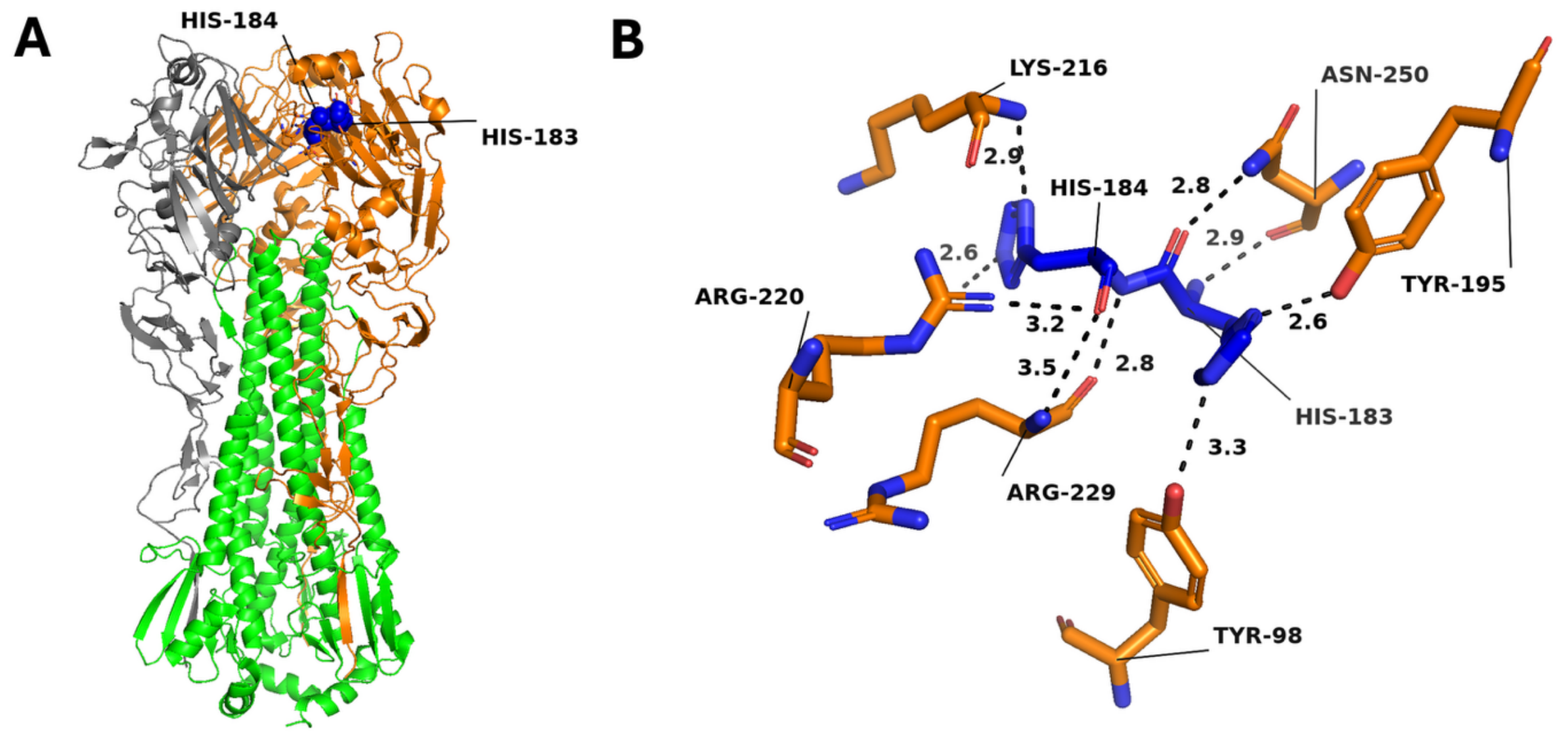

Figure S8. Network of polar interactions of His184 and His183. Localization (A) and interaction network (B) of His184 and His183For detailed explanation see text.

Mair et al. (2014)[12] investigated the HA1 subunit of H5 HA from both a highly pathogenic strain (A/chicken/Vietnam/P41/2005) and a low-pathogenic strain (A/teal/Germany/Wv632/2005), focusing on the role of His184. His184 is highly conserved across IAV subtypes (excluding H17 and H18. Structural analysis by Mair et al. (2014)[12] revealed that, for A/chicken/Vietnam/P41/2005, His184 is embedded in an extensive hydrogen bond network involving several residues. We identified a comparable network involving His184 and His183, organised through an extensive array of polar interactions at the HA1-HA1 interface. This network includes the residues His183, His184, Asn186, Lys216, Ala218, Arg220, Arg227, Gly228 and Asp231, with bond lengths ranging from 2.6 Å to 3.4 Å. Direct interactions were observed between His184 and Lys216, Arg220 and Asp231; and between His183 and Asn25, Tyr98, Tyr195 and Arg229 (see Figure S8). Mair et al. (2014)[12] have shown that mutations of His184 to Ala, Asn or Asp either lead to a shift in the pH value of the conformational change towards higher values (i.e. destabilisation of the conformation), or complete suppression of the conformational transition (by Arg)[94]. Mutations of other residues in the interaction network also influence the stability of the conformation. A typical feature of highly pathogenic H5 viruses is a positively charged amino acid in position 216 (here Lys), which interacts with His184. Low pathogenic viruses have a negatively charged residue in this position. Mair et al. demonstrated that mutations at position 216 lead to lower stability of the HA conformation at acidic pH when a negatively charged amino acid is present. Mair et al. (2014)[12] concluded from molecular modelling that double protonation of His184 at low pH is likely to disrupt the interaction between Arg220 and Asn210 in the neighbouring HA1 head domain (Figure S8) and that the associated destabilisation of the trimeric structure leads to a conformational change (for further details see Mair et al. (2014)[12]). Our analysis revealed that the interaction between His184 and Lys216 is disrupted at acidic pH. In any case, these results demonstrate that the protonation of His184 at low pH weakens its interaction with neighbouring residue and thus may serve as a trigger of the conformational change. These results are in line with those of Dadonaite et al. (2024)[4], who demonstrated that mutating Lys216 as well as other residues within the network, decreases rather than increases HA stability and viral entry. The importance of these amino acids interacting with the two His residues is emphasised by the

fact that they are highly conserved, for example Arg220 and Arg229. The prominent role of charged amino acids in the stability of ECD led to the suggestion that their substitution could play an important role in virus adaptation[6]. The high sensitivity to mutations as well as the high degree of conservation of His184, and its embedding in a network of contacts suggest that this residue - possibly also His183 - is a pH sensor that triggers the conformational change.

Figure S9

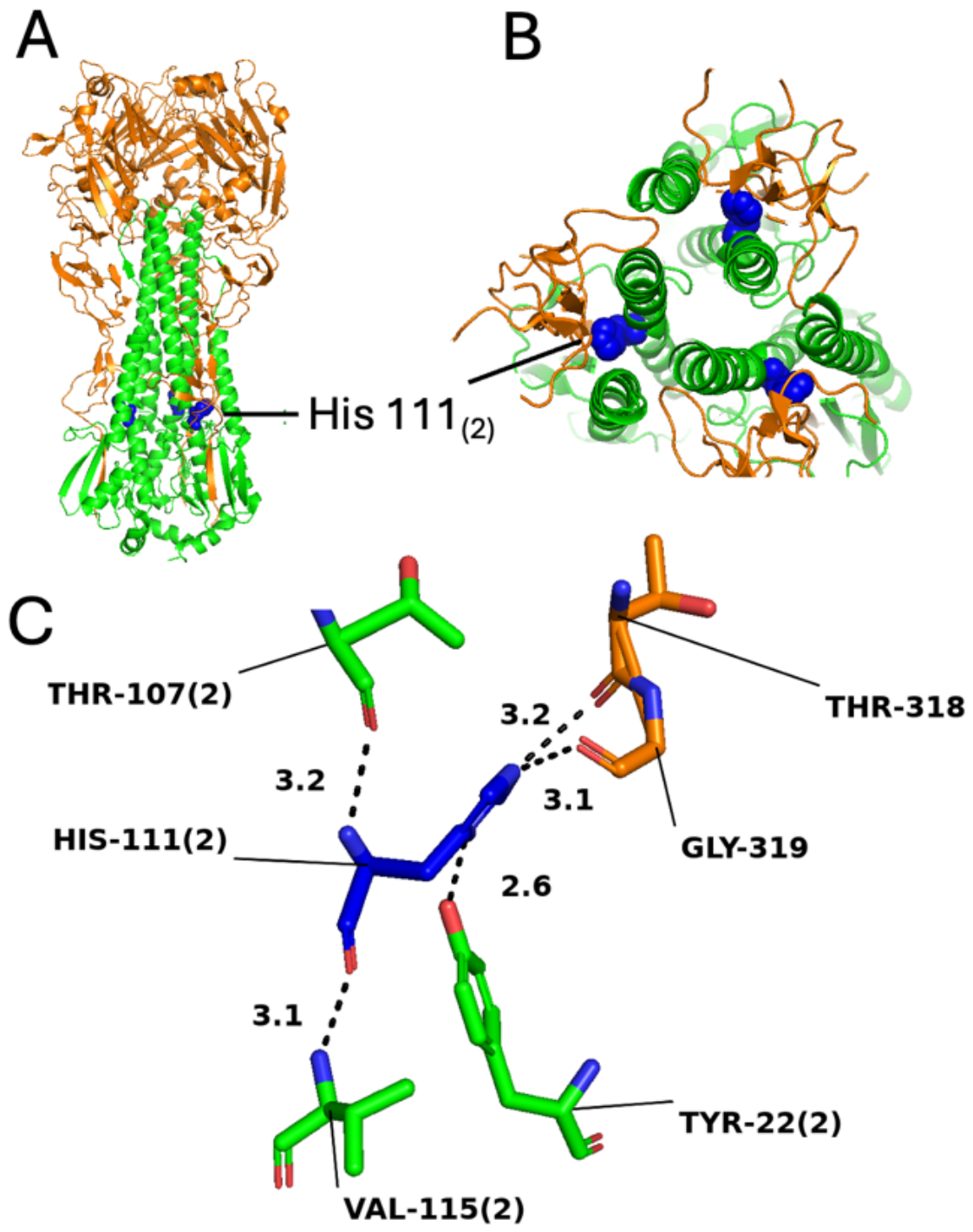

Figure S9. His111$_{(2)}$ interacts with several residues of the HA2 subunit and of the HA1 subunit of the monomer. In particular with the C terminal residue of the fusion peptide Tyr22$_{(2)}$. (A) Localisation of His111$_{(2)}$ in the stem region. (B) Top view on the localisation of His111$_{(2)}$. (C) Polar interactions of His111$_{(2)}$. For details see text.

His111$_{(2)}$: This residue is not exposed on the surface of the spike and is located close to the fusion peptide interacting with its C terminal residue Tyr22$_{(2)}$. It interacts also with Thr107$_{(2)}$, Leu108$_{(2)}$ and Val115$_{(2)}$ of the HA2 subunit. Furthermore, His111$_{(2)}$ establishes an interaction between the HA2 and HA1 subunits via contacts with Thr318 and Gly319 (Figure S9, for Tyr22$_{(2)}$ see also Figure S7)). Thus, this residue seems to be important for the stability of the ECD. Indeed, all mutations measured had a negative effect on the stability[4]. Based on the spatial positioning of the residue within the HA2 subunit, Mair et al. (2014)[12] suggested that it may contribute to later stages of the conformational change rather than its initiation. We assume that this conclusion also applies to His142$_{(2)}$. This residue is located in the ECD region close to the envelope, and it may be important for the later phase of conformational change, namely the formation of the six-helix bundle. Other studies have also suggested that the sequential protonation of His residues, such as His 111$_{(2)}$, as well as His18 and His38, controls the conformational change[13-15].

References

1 Thoennes, S. *et al.* Analysis of residues near the fusion peptide in the influenza hemagglutinin structure for roles in triggering membrane fusion. *Virology* **370**, 403–414 (2008). https://doi.org/10.1016/j.virol.2007.08.035

2 Reed, M. L. *et al.* Amino acid residues in the fusion peptide pocket regulate the pH of activation of the H5N1 influenza virus hemagglutinin protein. *Journal of virology* **83**, 3568–3580 (2009).

3 Mair, C. M., Ludwig, K., Herrmann, A. & Sieben, C. Receptor binding and pH stability — How influenza A virus hemagglutinin affects host-specific virus infection. *Biochimica et Biophysica Acta (BBA) - Biomembranes* **1838**, 1153–1168 (2014). https://doi.org/10.1016/j.bbamem.2013.10.004

4 Dadonaite, B. *et al.* Deep mutational scanning of H5 hemagglutinin to inform influenza virus surveillance. *PLOS Biology* **22**, e3002916 (2024). https://doi.org/10.1371/journal.pbio.3002916

5 Koerner, I., Matrosovich, M. N., Haller, O., Staeheli, P. & Kochs, G. Altered receptor specificity and fusion activity of the haemagglutinin contribute to high virulence of a mouse-adapted influenza A virus. *Journal of General Virology* **93**, 970–979 (2012). https://doi.org/´10.1099/vir.0.035782-0

6 Daniels, R. S. *et al.* Fusion mutants of the influenza virus hemagglutinin glycoprotein. *Cell* **40**, 431–439 (1985). https://doi.org/10.1016/0092-8674(85)90157-6

7 Korte, T., Epand, R. F., Epand, R. M. & Blumenthal, R. Role of the Glu Residues of the Influenza Hemagglutinin Fusion Peptide in the pH Dependence of Fusion Activity. *Virology* **289**, 353–361 (2001). https://doi.org/10.1006/viro.2001.1108

8 Langley, W. A. *et al.* Single residue deletions along the length of the influenza HA fusion peptide lead to inhibition of membrane fusion function. *Virology* **394**, 321–330 (2009). https://doi.org/10.1016/j.virol.2009.08.031

9 Zaraket, H., Bridges, O. A. & Russell, C. J. The pH of activation of the hemagglutinin protein regulates H5N1 influenza virus replication and pathogenesis in mice. *J Virol* **87**, 4826–4834 (2013). https://doi.org/10.1128/jvi.03110-12

10 Reed, M. L. *et al.* The pH of Activation of the Hemagglutinin Protein Regulates H5N1 Influenza Virus Pathogenicity and Transmissibility in Ducks. *Journal of Virology* **84**, 1527–1535 (2010). https://doi.org/10.1128/jvi.02069-09

11 Weis, W. I. *et al.* The structure of a membrane fusion mutant of the influenza virus haemagglutinin. *The EMBO Journal* **9**, 17–24 (1990). https://doi.org/10.1002/j.1460-2075.1990.tb08075.x

12 Mair, C. M. *et al.* A Histidine Residue of the Influenza Virus Hemagglutinin Controls the pH Dependence of the Conformational Change Mediating Membrane Fusion. *Journal of Virology* **88**, 13189–13200 (2014). https://doi.org/10.1128/jvi.01704-14

13 Mueller, D. S. *et al.* Histidine protonation and the activation of viral fusion proteins. *Biochemical Society Transactions* **36**, 43–45 (2008). https://doi.org/10.1042/bst0360043

14 Stevens, J. *et al.* Structure and Receptor Specificity of the Hemagglutinin from an H5N1 Influenza Virus. *Science* **312**, 404–410 (2006). https://doi.org/10.1126/science.1124513

15 Kampmann, T., Mueller, D. S., Mark, A. E., Young, P. R. & Kobe, B. The Role of Histidine Residues in Low-pH-Mediated Viral Membrane Fusion. *Structure* **14**, 1481–1487 (2006). https://doi.org/10.1016/j.str.2006.07.011
